# Localising individual atoms of tryptophan side chains in the metallo-*β*-lactamase IMP-1 by pseudocontact shifts from paramagnetic lanthanoid tags at multiple sites

**DOI:** 10.5194/mr-3-1-2022

**Published:** 2022-01-04

**Authors:** Henry W. Orton, Iresha D. Herath, Ansis Maleckis, Shereen Jabar, Monika Szabo, Bim Graham, Colum Breen, Lydia Topping, Stephen J. Butler, Gottfried Otting

**Affiliations:** 1 ARC Centre of Excellence for Innovations in Peptide & Protein Science, Research School of Chemistry, Australian National University, Canberra, ACT 2601, Australia; 2 Research School of Chemistry, The Australian National University, Sullivans Creek Road, Canberra ACT 2601, Australia; 3 Latvian Institute of Organic Synthesis, Aizkraukles 21, 1006 Riga, Latvia​​​​​​​; 4 Monash Institute of Pharmaceutical Sciences, Monash University, Parkville, VIC 3052, Australia; 5 Department of Chemistry, Loughborough University, Epinal Way, Loughborough, LE11 3TU, United Kingdom

## Abstract

The metallo- β -lactamase IMP-1 features a flexible loop near the active site that assumes different conformations in single crystal structures, which may assist in substrate binding and enzymatic activity. To probe the position of this loop, we labelled the tryptophan residues of IMP-1 with 7- ${}^{13}$ C-indole and the protein with lanthanoid tags at three different sites. The magnetic susceptibility anisotropy ( Δχ ) tensors were determined by measuring pseudocontact shifts (PCSs) of backbone amide protons. The Δχ tensors were subsequently used to identify the atomic coordinates of the tryptophan side chains in the protein. The PCSs were sufficient to determine the location of Trp28, which is in the active site loop targeted by our experiments, with high accuracy. Its average atomic coordinates showed barely significant changes in response to the inhibitor captopril. It was found that localisation spaces could be defined with better accuracy by including only the PCSs of a single paramagnetic lanthanoid ion for each tag and tagging site. The effect was attributed to the shallow angle with which PCS isosurfaces tend to intersect if generated by tags and tagging sites that are identical except for the paramagnetic lanthanoid ion.

## 1 Introduction

The metallo- β -lactamase IMP-1 is an enzyme that hydrolyses β -lactams, thus conferring penicillin resistance to bacteria. First identified 30 years ago in the Gram-negative bacteria in the early 1990s from *Pseudomonas aeruginosa* and *Serratia* *marcescens* (Bush, 2013), IMP-1 has become a serious clinical problem due to horizontal gene transfer by a highly mobile gene (*bla* ${}_{\text{IMP-1}}$ ) located on an integron (Arakawa et al., 1995), as the *bla* ${}_{\text{IMP-1}}$ gene has been detected in isolates of *Klebsiella pneumoniae*, *Pseudomonas putida*, *Alcaligenes xylosoxidans*, *Acinetobacter junii*, *Providencia rettgeri*, *Acinetobacter baumannii* and *Enterobacter aerogenes* (Ito et al., 1995; Laraki et al., 1999a; Watanabe et al., 1991). Critically, IMP-1 also confers resistance to recent generations of carbapenems and extended-spectrum cephalosporins (Laraki et al., 1999b; Bush, 2010; van Duin et al., 2013).

**Figure 1:**
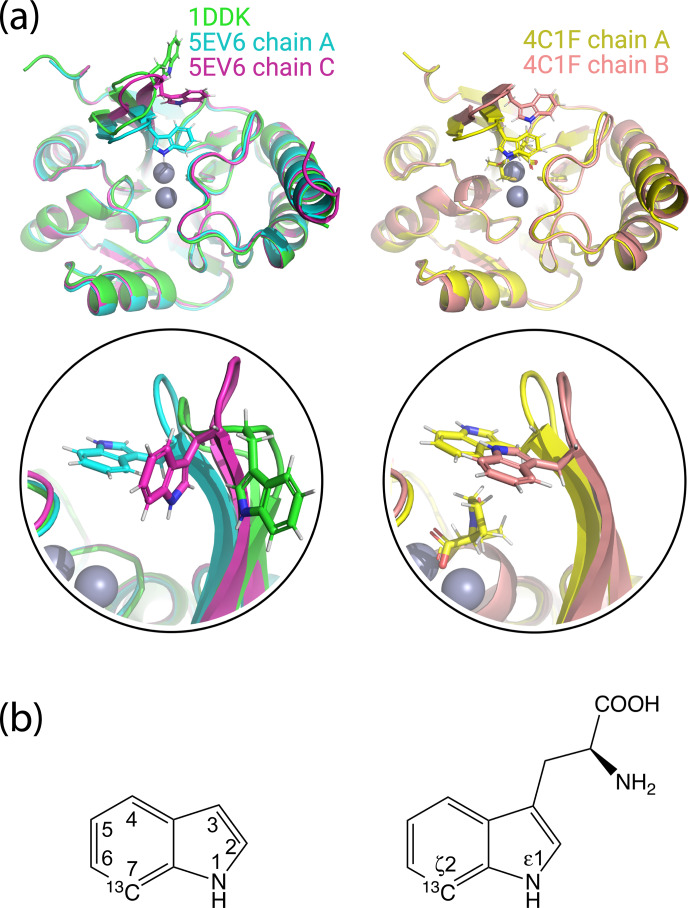
Crystal structures of IMP-1 with different conformations of the loop L3 and chemical structures of indole and tryptophan with atom names. **(a)** Superimposition of crystal structures of IMP-1 highlighting structural variations of Trp28 and the associated loop L3. The structures shown are of the Zn ${}^{2+}$ /Zn ${}^{2+}$ complex without an inhibitor (green, PDB ID 1DDK, Concha et al., 2000; cyan for chain A and magenta for chain C, PDB ID 5EV6, Hinchliffe et al., 2016), with bound L-captopril (yellow for chain A and salmon for chain B, PDB ID 4CIF, Brem et al., 2016). Zn ${}^{2+}$ ions are represented by grey spheres and bound captopril is shown in the structure 4C1F chain A. **(b)** Chemical structures of indole and tryptophan with selected ring positions labelled according to IUPAC conventions. The present work used indole synthesised with a ${}^{13}$ C- ${}^{1}$ H group in position 7 and deuterium in ring positions 2, 3, 4, 5 and 6 (Maleckis et al., 2021).

Multiple crystal structures have been solved of IMP-1, free and in complexes with various inhibitors (Concha et al., 2000; Toney et al., 2001; Moali et al., 2003; Hiraiwa et al., 2014; Brem et al., 2016; Hinchliffe et al., 2016, 2018; Wachino et al., 2019; Rossi et al., 2021). IMP-1 belongs to subclass B1 of metallo- β -lactamases, which contain two zinc ions bridged by the sulfur atom of a cysteine residue in the active site (Concha et al., 2000). One of the Zn ${}^{2+}$ ions can readily be replaced by a Fe ${}^{3+}$ ion (Carruthers et al., 2014). The active site is flanked by a loop (referred to as loop L3) that contains a highly solvent-exposed tryptophan residue surrounded by glycine residues on either side. Both the loop and the tryptophan residue (Trp28 in the IMP-1-specific numbering used by Concha et al., 2000, and Trp64 in the universal numbering scheme by Galleni et al., 2001) assume different conformations in different crystal structures, suggesting that the loop acts as a mobile flap to cover bound substrate (Fig. 1a). The L3 loop and the functional implication of its flexibility have been studied extensively for different metallo- β -lactamases containing the Gly–Trp–Gly motif in the loop (Huntley et al., 2000, 2003; Moali et al., 2003; Yamaguchi et al., 2015; Palacios et al., 2019; Gianquinto et al., 2020; Softley et al., 2020). Flexibility of the L3 loop is a general feature also of many metallo- β -lactamases without the Gly–Trp–Gly motif and is thought to contribute to the wide range of β -lactam substrates that can be hydrolysed by the enzymes (González et al., 2016; Linciano et al., 2019; Salimraj et al., 2018). In the case of the metallo- β -lactamase from *B. fragilis*, which is closely related to IMP-1, electron density could be detected for the Gly–Trp–Gly motif in the crystal structure of the protein in the presence (Payne et al., 2002) but not absence of an inhibitor (Concha et al., 1996), and an NMR relaxation study in solution confirmed the increased flexibility of both the L3 loop and, in particular, the side chain of the tryptophan residue (Huntley et al., 2000). A similar situation prevails in the case of the IMP-1 variant IMP-13, where different crystal structures of the ligand-free protein show the L3 loop in very different conformations, sometimes lacking electron density, while NMR relaxation measurements confirmed the increased flexibility of the loop (Softley et al., 2020).

Due to the rigidity of their side chains, tryptophan residues frequently contribute to the structural stability of 3D protein folds, and it is unusual to observe tryptophan side chains fully solvent-exposed as in the Gly–Trp–Gly motif of substrate-free IMP-1. The functional role of Trp28 in IMP-1 was assessed in an early mutation study by mutating Trp28 to alanine and, in a different experiment, eliminating the L3 loop altogether. Enzymatic activity measurements revealed an increase in the Michaelis constant $K_{m}$ and a decrease in $k_{\mathrm{cat}}/K_{m}$ ratios for all β -lactams tested, illustrating the importance of the Trp28 side chain for catalytic activity. Complete removal of the L3 loop reduced the $k_{\mathrm{cat}}/K_{m}$ ratios even further but without completely abolishing the enzymatic activity (Moali et al., 2003).

In the crystalline state, the conformation of a solvent-exposed loop is easily impacted by crystal packing forces. Therefore, it is unclear what the actual conformation of the L3 loop is in solution. To address this question, we used solution NMR spectroscopy to assess the location of Trp28 in IMP-1 both in the absence and presence of the inhibitor L-captopril, which inhibits metallo- β -lactamases by binding to the active-site zinc ions (Brem et al., 2016). The analysis was hindered by incomplete backbone resonance assignments of IMP-1 attributed to conformational exchange processes in parts of the protein (Carruthers et al., 2014). As it is difficult to accurately position the atoms of a solvent-exposed polypeptide loop in solution by nuclear Overhauser effects (NOE), we used pseudocontact shifts (PCSs) generated by lanthanoid ions attached at different sites of IMP-1 to determine the location of Trp28 relative to the core of the protein. PCSs generated by multiple different paramagnetic metal ions or the same metal ion attached at different sites of a protein have previously been shown to allow localising atoms at remote sites of interest, such as in specific amino acid side chains (Pearce et al., 2017; Lescanne et al., 2018), bound ligand molecules (Guan et al., 2013; Chen et al., 2016) or proteins (Pintacuda et al., 2006; Keizers et al., 2010; de la Cruz et al., 2011; Kobashigawa et al., 2012; Brewer et al., 2015) or full 3D structure determinations of proteins (Yagi et al., 2013; Crick et al., 2015; Pilla et al., 2017).

IMP-1 contains six tryptophan residues, each containing several aromatic hydrogens with similar chemical shifts. To increase the spectral resolution in the 2D NMR spectra recorded for PCS measurements, we labelled each tryptophan side chain with a single ${}^{13}$ C atom by expressing the protein in the presence of 7- ${}^{13}$ C-indole (Fig. 1b; Maleckis et al., 2021). The results show that the localisation spaces defined by the tryptophan PCSs fully agree with previously determined crystal structures of IMP-1 for all tryptophan residues. They suggest little change in the average conformation of the L3 loop upon binding of captopril. The results illustrate the accuracy with which the positions of individual atoms can be determined by PCSs from lanthanoid tags even in proteins of limited stability.

## 2 Experimental procedures

### 2.1 Production, purification and tagging of proteins

#### 2.1.1 Plasmid constructs and ${}^{13}$ C-labelled indole

Three different cysteine mutations (A53C, N172C and S204C) were introduced into the *bla* ${}_{\text{IMP1}}$ gene in the pET-47b( + ) plasmid using a modified QuikChange protocol (Qi and Otting, 2019).

Deuterated 7- ${}^{13}$ C-indole was synthesised as described with deuteration in all positions other than position 7 (Maleckis et al., 2021). The amino acid sequence of the protein was that reported in the crystal structure 4UAM (Carruthers et al., 2014), except that the N-terminal alanine residue was substituted by a methionine to avoid heterogeneity by incomplete processing by amino peptidase.

#### 2.1.2 Protein production

Uniformly ${}^{15}$ N-labelled samples of the cysteine mutants of IMP-1 were expressed in *E. coli* BL21(DE3) cells. The cells were grown at 37 ${}^{\circ }$ C in Luria–Bertani (LB) medium containing 50 mg L ${}^{-1}$ kanamycin until the OD ${}_{600}$ reached 0.6–0.8 and were then transferred to 300 mL of M9 medium (6 g L ${}^{-1}$ Na ${}_{2}$ HPO ${}_{4}$ , 3 g L ${}^{-1}$ KH ${}_{2}$ PO ${}_{4}$ , 0.5 g L ${}^{-1}$ NaCl, pH 7.2) supplemented with 1 g L ${}^{-1}$ of ${}^{15}$ NH ${}_{4}$ Cl. After induction with isopropyl- β -D-thiogalactopyranoside (IPTG, final concentration 1 mM), the cells were incubated at room temperature for 16 h. Following centrifugation, the cells were resuspended in buffer A (50 mM HEPES, pH 7.5, 100 µ M ZnSO ${}_{4}$ ) for lysis by a homogeniser (Avestin Emulsiflex C5). The supernatant of the centrifuged cell lysate was loaded onto a 5 mL SP column, the column was washed with 20 column volumes buffer B (same as buffer A but with 50 mM NaCl) and the protein was eluted with a gradient of buffer C (same as buffer A but with 1 M NaCl).

IMP-1 samples containing 7- ${}^{13}$ C-tryptophan were produced by continuous-exchange cell-free protein synthesis (CFPS) from PCR-amplified DNA with eight-nucleotide single-stranded overhangs as described (Wu et al., 2007), using 7- ${}^{13}$ C-indole as a precursor for the in vitro production of tryptophan (Maleckis et al., 2021). The CFPS reactions were conducted at 30 ${}^{\circ }$ C for 16 h using 1 mL inner reaction mixture and 10 mL outer buffer. Tryptophan was omitted from the mixture of amino acids provided and deuterated 7- ${}^{13}$ C-indole was added from a stock solution in 50 % DMSO/50 % H ${}_{2}$ O to the inner and outer buffers at a final concentration of 0.75 mM. The protein samples were purified as described above. About 5 mg of the indole was required for preparing each NMR sample.

#### 2.1.3 Ligation with C2-Ln ${}^{3+}$ tag

To ensure the reduced state of cysteine thiol groups, the protein samples were treated with 2 mM dithiothreitol (DTT) for 1 h. Subsequently, the DTT was removed using an Amicon ultrafiltration centrifugal tube with a molecular weight cut-off of 10 kDa, concentrating the protein samples to 50 µ M in buffer A. The samples were incubated overnight at room temperature with shaking in the presence of 5-fold molar excess of C2 tag (Graham et al., 2011; de la Cruz et al., 2011) loaded with either Y ${}^{3+}$ , Tb ${}^{3+}$ or Tm ${}^{3+}$ . Following the tagging reaction, the samples were washed using an Amicon centrifugal filter unit to remove unbound tag and the buffer was exchanged to the NMR buffer (20 mM MES, pH 6.5, 100 mM NaCl).

#### 2.1.4 Ligation with a C12-Ln ${}^{3+}$ tag

The ligation reaction of IMP-1 N172C with the C12-Ln ${}^{3+}$ tag loaded with either Y ${}^{3+}$ , Tb ${}^{3+}$ or Tm ${}^{3+}$ (Herath et al., 2021) was conducted in the same way as with the C2-Ln ${}^{3+}$ tags, except that the reactions were carried out in buffer A with the pH adjusted to 7.0.

### 2.2 NMR spectroscopy

All NMR data were acquired at 37 ${}^{\circ }$ C on Bruker 600 and 800 MHz NMR spectrometers equipped with TCI cryoprobes designed for 5 mm NMR tubes, but only 3 mm NMR tubes were used in this project. Protein concentrations were 0.6 and 0.2 mM for ${}^{15}$ N-HSQC spectra of samples labelled with the C2 and C12 tags, respectively. The protein concentrations were 0.4 mM for ${}^{13}$ C-HSQC and NOE-relayed ${}^{13}$ C-HSQC spectra. ${}^{15}$ N-HSQC spectra were recorded at a ${}^{1}$ H-NMR frequency of 800 MHz with $t_{\text{1max}}=40$ ms and $t_{\text{2max}}=170$ ms, using a total recording time of 3 h per spectrum. ${}^{13}$ C-HSQC spectra were recorded with the S ${}^{3}$ E filter to select the low-field doublet component due to the ${}^{1}J_{\text{HC}}$ coupling of the ${}^{13}$ C-labelled tryptophan side chains. The pulse sequence is shown in Fig. S9 in the Supplement and the spectra were recorded at a ${}^{1}$ H-NMR frequency of 600 MHz using $t_{\text{1max}}=20$ –50 ms, $t_{\text{2max}}=106$ ms and total recording times of 2 h per spectrum. ${}^{13}$ C-HSQC spectra with NOE relay were recorded without decoupling in the ${}^{13}$ C dimension, relying on relaxation and ${}^{13}$ C equilibrium magnetisation to emphasise the narrow doublet component. The NOE mixing time was 150 ms and the total recording time 3 h per spectrum. The pulse sequence is shown in Fig. S10.

To account for uncertainties in concentration measurements, samples with L-captopril were prepared with a nominal ratio of captopril to protein of 1.5 : 1. In the case of samples tagged with the C2 tag, however, this led to gradual release of some of the tag, as captopril contains a free thiol group and the disulfide linkage of the C2 tag is sensitive to chemical reduction. To limit this mode of sample degradation, the NOE-relayed [ ${}^{13}$ C, ${}^{1}$ H]-HSQC spectra were recorded with a smaller excess of captopril.

### 2.3 Δχ -tensor fits

The experimental PCSs ( $\Delta \delta^{\text{PCS}}$ ) were measured in parts per million (ppm) as the amide proton chemical shift observed in NMR spectra recorded for the IMP-1 mutants A53C, N172C and S204C tagged with Tm ${}^{3+}$ or Tb ${}^{3+}$ tags minus the corresponding chemical shift measured for samples made with Y ${}^{3+}$ tags. The resonance assignments of the wild-type Zn ${}_{2}$ enzyme (BMRB entry 25063) were used to assign the ${}^{15}$ N-HSQC cross-peaks in the diamagnetic state. The program Paramagpy (Orton et al., 2020) was used to fit magnetic susceptibility anisotropy ( Δχ ) tensors to crystal structures of IMP-1 solved in the absence and presence of the inhibitor captopril.

## 3 Results

### 3.1 Protein production

Three cysteine mutants of uniformly ${}^{15}$ N-labelled IMP-1 were produced in vivo, where cysteine residues replaced Ala53, Asn172 and Ser204, respectively. The purified proteins were tagged with C2 tags containing Tb ${}^{3+}$ or Tm ${}^{3+}$ as the paramagnetic ions and Y ${}^{3+}$ as the diamagnetic reference. Samples of the uniformly ${}^{15}$ N-labelled mutant N172C were also ligated with C12 tags containing the same set of metal ions. The chemical structures of the tags are depicted in Fig. S1. To record ${}^{13}$ C- ${}^{1}$ H correlation spectra of the tryptophan side chains with minimal spectral overlap, additional samples of the cysteine mutants were produced with selectively ${}^{13}$ C-labelled tryptophan residues. These samples were produced by cell-free protein synthesis in the presence of 7- ${}^{13}$ C indole, deuterated except at the 7 position, with the omission of tryptophan, using a recently established protocol (Maleckis et al., 2021). The residual activity of tryptophan synthase in the cell-free extract was sufficient to produce tryptophan from the added ${}^{13}$ C-labelled indole. The resulting tryptophan residues contained a ${}^{13}$ C- ${}^{1}$ H group in position 7 ( ${}^{13}$ C ${}^{\zeta 2}$ and ${}^{1}$ H ${}^{\zeta 2}$ in IUPAC nomenclature; Markley et al., 1998) and deuterons at all other hydrogen positions of the indole ring except for the H ${}^{\text{N}}$ atom (H ${}^{\varepsilon 1}$ in IUPAC nomenclature). The cell-free expression yielded about 2 mg of purified protein per millilitre of inner cell-free reaction mixture. Mass spectrometry indicated that the tryptophan residues of IMP-1 were ${}^{13}$ C / ${}^{2}$ H-labelled with about 80 % labelling efficiency at each of the six tryptophan positions (Fig. S2). The purified proteins were ligated with C2-Ln ${}^{3+}$ tags containing either Tb ${}^{3+}$ , Tm ${}^{3+}$ or Y ${}^{3+}$ as in the case of the ${}^{15}$ N-labelled samples. Ligation yields with the C2 tags were practically complete, as indicated by mass spectrometry (Fig. S2). The ligation yield of the N172C mutant with C12 tags was about 90 % (Herath et al., 2021).

### 3.2 NMR experiments and resonance assignments

[ ${}^{15}$ N, ${}^{1}$ H]-HSQC spectra were measured of the tagged proteins in the free state and in the presence of L-captopril (Figs. S3–S8). ${}^{1}$ H PCSs of backbone amide protons measured in these spectra were used to establish the Δχ tensors relative to the protein. The resonance assignment of the [ ${}^{15}$ N, ${}^{1}$ H]-HSQC spectra in the presence of inhibitor was transferred from the corresponding spectra recorded in the absence of inhibitor. As no resonance assignments could reliably be made in this way in areas of spectral overlap, fewer resonance assignments were available in the presence than absence of inhibitor. Furthermore, due to captopril releasing some of the C2 tags from the protein by breaking the disulfide bridge of the tag attachment, spectra recorded in the presence of captopril contained additional cross-peaks from diamagnetic protein.

To obtain tagged protein that is inert against chemical reduction, we also attached the C12 tag to the mutant N172C. This tag, however, caused the appearance of additional peaks in the [ ${}^{15}$ N, ${}^{1}$ H]-HSQC spectra (Fig. S7). The additional peaks appeared in different sample preparations, indicating sample degradation or perturbation of the local protein structure by the tag. We therefore based the rest of the work mainly on the PCSs obtained with the C2 tags. Tables S1 and S2 list the PCSs of the backbone amides measured in the absence and presence of captopril.

**Figure 2:**
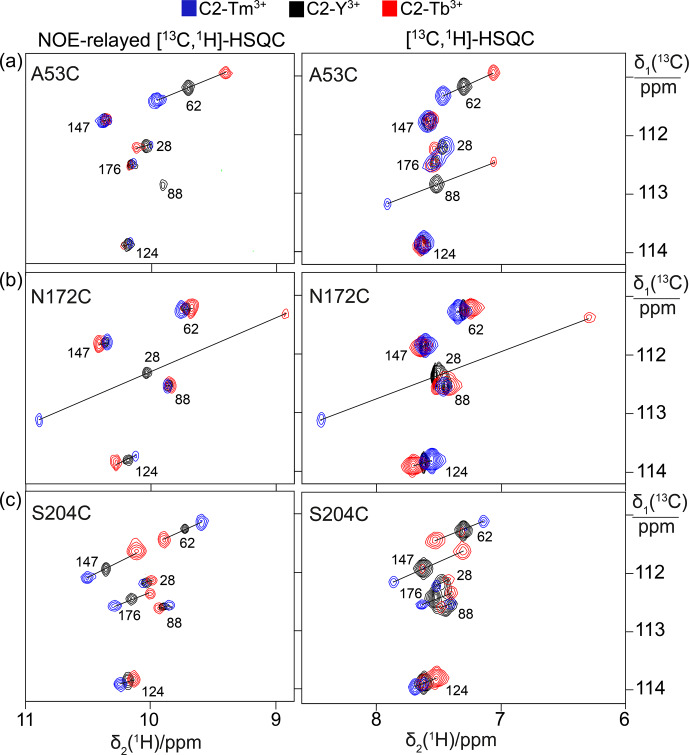
PCSs observed in ${}^{13}$ C- ${}^{1}$ H correlation spectra of 0.4 mM solutions of IMP-1 mutants tagged with C2-Ln ${}^{3+}$ tags and containing selectively isotope-labelled tryptophan produced from 7- ${}^{13}$ C-indole deuterated in positions 2, 4, 5 and 6. The plots show superimpositions of spectra recorded with diamagnetic (C2-Y ${}^{3+}$ , black) or paramagnetic (C2-Tb ${}^{3+}$ , red; C2-Tm ${}^{3+}$ , blue) tags. All spectra were recorded with spin-state selection in the ${}^{13}$ C dimension to record the narrow low-field component of each ${}^{13}$ C doublet. Right panels: [ ${}^{13}$ C, ${}^{1}$ H]-HSQC spectra. Left panels: NOE-relayed [ ${}^{13}$ C, ${}^{1}$ H]-HSQC spectra (150 ms NOE mixing time) to record the H ${}^{\varepsilon 1}$ resonances of the tryptophan side chains. PCSs are indicated by lines connecting the peaks of paramagnetic and diamagnetic samples. The cross-peaks are labelled with the residue number of the individual tryptophan residues. **(a)** Mutant A53C. **(b)** Mutant N172C. **(c)** Mutant S204C.​​​​​​​

${}^{1}$ H PCSs of the tryptophan H ${}^{\zeta 2}$ protons were measured in [ ${}^{13}$ C, ${}^{1}$ H]-HSQC spectra recorded with the S ${}^{3}$ E spin-state selection element (Meissner et al., 1997) in the ${}^{13}$ C dimension to select the slowly relaxing components of the doublets split by ${}^{1}J_{\text{HC}}$ couplings. Cross-peaks were observed for all six tryptophan residues except for the mutant N172C, which displayed cross-peaks of only five tryptophan indoles (Fig. 2). The missing signal was attributed to Trp176 because of its close proximity to the tagging site. The indole H ${}^{\varepsilon 1}$ proton is located within 2.9 Å of the H ${}^{\zeta 2}$ proton and the NOE between both protons was readily observed in a [ ${}^{13}$ C, ${}^{1}$ H]-HSQC experiment with NOE relay (Fig. 2). The H ${}^{\varepsilon 1}$ chemical shifts afforded better spectral resolution than the H ${}^{\zeta 2}$ resonances. Comparison of the predicted and observed PCSs yielded resonance assignments of all tryptophan H ${}^{\varepsilon 1}$ cross-peaks with particular clarity in the NOE-relayed [ ${}^{13}$ C, ${}^{1}$ H]-HSQC spectrum (Fig. 2). In addition, the assignment was supported by paramagnetic relaxation enhancements (for example, Trp88 is near residue 53 and therefore its cross-peaks were strongly attenuated in the paramagnetic samples of the A53C mutant). Different PCSs were observed for all six tryptophan side chains and different PCSs were observed for the H ${}^{\zeta 2}$ and H ${}^{\varepsilon 1}$ protons within the same indole side chain. Each of the tryptophan side chains showed PCSs in most, if not all, of the mutants. As the L3 loop is near residue 172, the mutant N172C endowed Trp28 with particularly large PCSs. Tables S3 and S4 report the PCSs measured in this way for the samples labelled with C2 tags.

In contrast, assigning the indole N–H groups in the [ ${}^{15}$ N, ${}^{1}$ H]-HSQC spectra was much more difficult because IMP-1 is a protein prone to showing more than a single peak per proton (Figs. S5 and S6). In particular, the [ ${}^{15}$ N, ${}^{1}$ H]-HSQC spectrum of wild-type IMP-1 selectively labelled with ${}^{15}$ N-tryptophan displayed six intense and at least three weak N ${}^{\varepsilon 1}$ –H ${}^{\varepsilon 1}$ cross-peaks (Fig. S6; Carruthers et al., 2014) and the [ ${}^{15}$ N, ${}^{1}$ H]-HSQC spectra of the tagged cysteine mutants showed evidence of heterogeneity too (Fig. S5). Nonetheless, the six most intense N ${}^{\varepsilon 1}$ –H ${}^{\varepsilon 1}$ cross-peaks could be assigned by comparison to the PCSs observed in the NOE-relayed [ ${}^{13}$ C, ${}^{1}$ H]-HSQC spectrum, and this assignment was used to measure the PCSs of the tryptophan H ${}^{\varepsilon 1}$ resonances in the mutant N172C tagged with the C12 tag (Fig. S8; Table S4).

**Figure 3:**
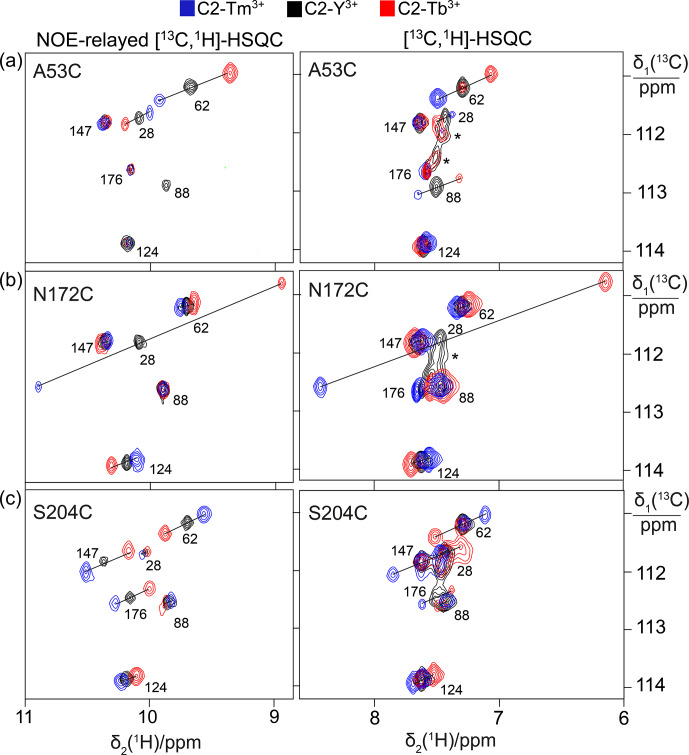
Effect of the presence of L-captopril on the PCSs observed in ${}^{13}$ C- ${}^{1}$ H correlation spectra of 0.4 mM solutions of IMP-1 mutants. Protein preparations and experimental parameters were the same as in Fig. 2. Spectra recorded with diamagnetic (C2-Y ${}^{3+}$ , black) or paramagnetic (C2-Tb ${}^{3+}$ , red; C2-Tm ${}^{3+}$ , blue) tags are superimposed. Right column: [ ${}^{13}$ C, ${}^{1}$ H]-HSQC spectra. Left column: NOE-relayed [ ${}^{13}$ C, ${}^{1}$ H]-HSQC spectra recorded with 150 ms NOE mixing time. Stars mark cross-peaks of species putatively attributed to protein degradation. **(a)** Mutant A53C. **(b)** Mutant N172C. **(c)** Mutant S204C.

Spectra recorded in the presence of L-captopril were very similar to those recorded without the inhibitor, except that some new, narrow C–H cross-peaks appeared in the [ ${}^{13}$ C, ${}^{1}$ H]-HSQC spectra of mutants A53C and S204C, which were suggestive of protein degradation (Fig. 3). We consequently used the better-resolved indole N–H cross-peaks to identify the correct parent C–H cross-peaks. The chemical shifts of the tryptophan side chains changed very little in response to the presence of L-captopril, except for the ${}^{13}$ C-chemical shift of Trp28, which is nearest to the ligand binding site. The PCSs of the indole protons measured in the presence of the inhibitor are listed in Tables S5 and S6.

### 3.3 Δχ -tensor fits

**Figure 4:**
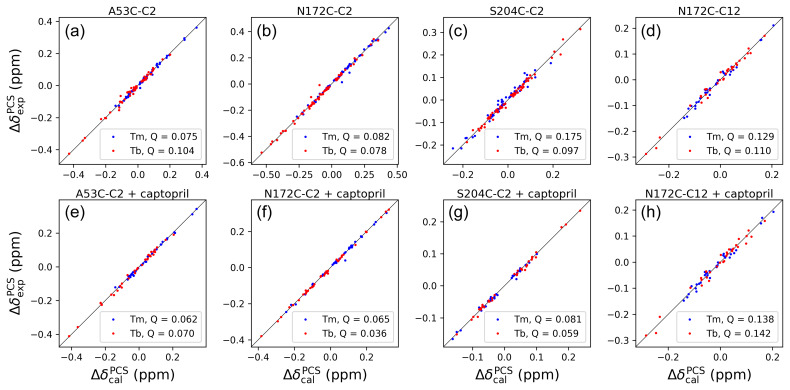
Correlations between back-calculated and experimental ${}^{1}$ H PCSs measured of backbone amides of IMP-1 with C2 tags at three different sites (positions 53, 172 and 204) and the C12 tag in position 172. Red and blue data points correspond to the PCS data obtained with Tb ${}^{3+}$ and Tm ${}^{3+}$ tags, respectively. **(a)** Mutant A53C with C2 tag. **(b)** Mutant N172C with C2 tag. **(c)** Mutant S204C with C2 tag. **(d)** Mutant N172C with C12 tag. **(e)** Same as **(a)** but in the presence of captopril. **(f)** Same as **(b)** but in the presence of captopril. **(g)** Same as **(c)** but in the presence of captopril. **(h)** Same as **(d)** but in the presence of captopril. PCS data in **(a)**–**(d)** were used to fit Δχ tensors to the structure 5EV6. PCS data in **(e)**–**(f)** were used to fit Δχ tensors to the structure 4C1F.

The Δχ -tensor parameters were determined using the program Paramagpy (Orton et al., 2020) using all available ${}^{1}$ H PCSs measured of backbone amides. Comparing the Δχ -tensor fits to the crystal structures 5EV6 chains A and C (Hinchliffe et al., 2016) and 1DDK (Concha et al., 2000) of the free protein, chain A of the structure 5EV6 proved to produce the smallest Q factor by a small margin (Fig. S11) and was used as the reference structure of the free protein for the subsequent evaluation. Similarly, chain A of the co-crystal structure published with the inhibitor L-captopril (PDB ID: 4C1F; Brem et al., 2016) on average delivered better fits than chain B and was used as the reference structure for the NMR data recorded in the presence of L-captopril. The Δχ -tensor fits of each mutant and tag used a common metal position for the data obtained with the Tb ${}^{3+}$ and Tm ${}^{3+}$ tags. The fits positioned the paramagnetic centres at distances between 8.2 and 9.4 Å from the C ${}^{\beta }$ atom of the tagged cysteine residues, which is compatible with the chemical structure of the C2 tag. Figure 4 shows the correlations between back-calculated and experimental PCSs and Tables S7 and S8 report the fitted Δχ -tensor parameters. Very similar Q factors were obtained when using the PCSs measured in the absence of inhibitor to fit the Δχ tensor to the co-crystal structure 4C1F or the PCSs measured in the presence of inhibitor to fit the Δχ tensor to the crystal structure of the free protein. This indicates that the protein structure did not change very much in response to inhibitor binding. This conclusion was also indicated by the similarity between the backbone PCSs observed with and without inhibitor (Fig. S12).

The Δχ tensors obtained with the Tb ${}^{3+}$ tags were larger than those obtained with the Tm ${}^{3+}$ tags, which is also reflected by the consistently larger PCSs observed in the ${}^{13}$ C- ${}^{1}$ H correlation spectra of Figs. 2 and 3. The fits of Δχ tensors to the protein backbone also yielded better Q factors for PCSs generated by Tb ${}^{3+}$ than Tm ${}^{3+}$ ions. Therefore, we determined the localisation spaces of the tryptophan side chains in the first instance by using their ${}^{1}$ H PCSs measured with Tb ${}^{3+}$ tags only.

### 3.4 Determining the localisation spaces of tryptophan side chains

**Figure 5:**
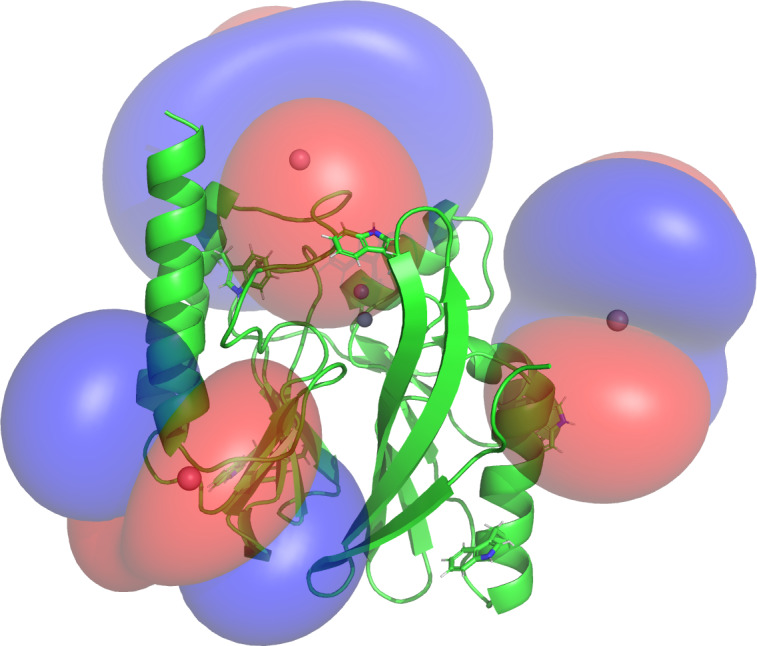
PCS isosurfaces of IMP-1 mutants A53C, N172C and S204C plotted on the crystal structure 5EV6. The respective Δχ tensors were determined from the ${}^{1}$ H PCSs measured of backbone amides. Blue/red isosurfaces correspond to PCSs of ± 1.0 ppm, respectively, generated with C2-Tb ${}^{3+}$ tags.

The Δχ tensors determined from backbone amides not only enabled the resonance assignment of the tryptophan side chains by comparing back-calculated with experimental PCSs, but also allowed translation of the indole PCSs into restraints that define the locations of the tryptophan H ${}^{\zeta 2}$ and H ${}^{\varepsilon 1}$ atoms with respect to the rest of the protein. The concept of localising nuclear spins by PCSs that are generated by lanthanoid tags at different sites is well established (see, e.g., Yagi et al., 2013; Lescanne et al., 2018; Zimmermann et al., 2019). It can be visualised by representing each PCS restraint by the corresponding PCS isosurface, which comprises all points in space where this PCS value is generated by the Δχ tensor (Fig. 5). With PCS restraints from two different metal sites, the intersection between the respective isosurfaces defines a line. The intersection of this line with the PCS isosurface from a third Δχ tensor defines two points. While a fourth Δχ tensor could unambiguously produce a single solution, a fourth tensor may not be required if one of these two points is incompatible with the covalent structure of the protein. Under favourable circumstances, the constraints imposed by the covalent structure may even allow the accurate positioning of nuclear spins by PCSs generated from only two different Δχ tensors (Pearce et al., 2017). Therefore, the present study was successful with only three different tagging sites. Figure S13 illustrates the concept for the Trp28 H ${}^{\varepsilon 1}$ atom.

**Figure 6:**
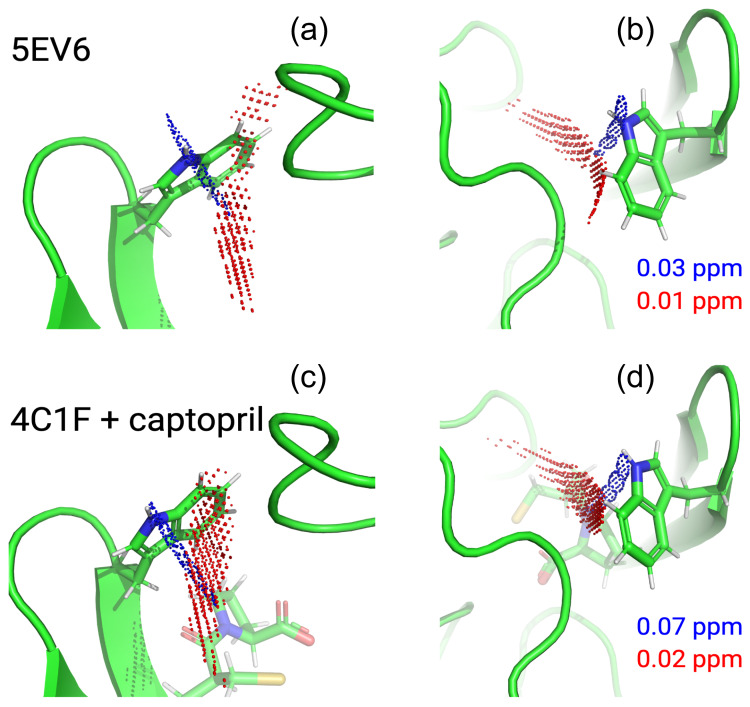
Localisation space of the side chain of Trp28 defined by the PCSs from tags in IMP-1 mutants A53C, N172C and S204C. The left and right panels display the same results in two different orientations. Red and blue points outline localisation spaces determined for the H ${}^{\zeta 2}$ and H ${}^{\varepsilon 1}$ atoms, respectively. The localisation space of the H ${}^{\zeta 2}$ atom was defined by the PCSs and Δχ tensors determined for the Tb ${}^{3+}$ -loaded C2 tags, while the localisation space of the H ${}^{\varepsilon 1}$ atom was restricted by additional data obtained with the C12-Tb ${}^{3+}$ tag at site N172C. The boundaries of the respective localisation spaces displayed are defined by the PCS RMSD values indicated in parts per million. The top panel depicts the localisation spaces determined for the free protein plotted on chain A of the crystal structure 5EV6 depicted in two different orientations. The lower panel depicts the localisation spaces determined in the presence of captopril plotted on chain A of the crystal structure 4C1F.

The spatial definition of the intersection point defined by the PCS isosurfaces depends on the experimental uncertainties in a non-isotropic way, as the PCS isosurfaces rarely intersect in an orthogonal manner and the PCS gradients differ for each Δχ tensor. To capture a localisation space, which allows for the experimental uncertainty in the measured PCS data and fitted Δχ tensors, we mapped the spatial field of root-mean-squared deviations (RMSDs) between experimental and calculated PCS values and defined the boundary of the localisation space by a maximal RMSD value. In addition, uncertainties in the Δχ tensors were propagated by averaging over the results from 20 Δχ -tensor fits performed with random omission of 20 % of the backbone PCS data. In the present work, the routine for defining the localisation space was implemented as a script in the software Paramagpy (Orton et al., 2020). Figure 6 shows the resulting localisation spaces for the H ${}^{\varepsilon 1}$ and H ${}^{\zeta 2}$ atoms of Trp28 using the PCS data obtained for the three cysteine mutants A53C, N172C and S204C with the C2-Tb ${}^{3+}$ tag as well as the N172C mutant with the C12-Tb ${}^{3+}$ tag.

The localisation spaces found for the H ${}^{\varepsilon 1}$ and H ${}^{\zeta 2}$ atoms of Trp28 were clearly different. Furthermore, the distance between them corresponded closely to the distance expected from the chemical structure of the indole ring (2.9 Å). The irregular shapes of the localisation spaces displayed in Fig. 6 purely reflect the relative geometry of the intersecting PCS isosurfaces and do not take into account any dynamic flexibility of the L3 loop or protein structure. In particular, the relevant PCS isosurfaces associated with the C2 tag at sites N172C and S204C intersect at a shallow angle, which leads to the elongated shape of the localisation space for the Trp28 H ${}^{\zeta 2}$ atom (Fig. S13). For the nitrogen-bound H ${}^{\varepsilon 1}$ atom, the localisation space was restricted further by the additional data obtained with the C12 tag at site N172C (Fig. 6). Calculating the localisation spaces from the Tm ${}^{3+}$ data yielded very similar results (Fig. S14). The agreement of the localisation spaces of Trp28 with chain A of the previously published crystal structure 5EV6 is excellent, and they are clearly incompatible with the conformations observed in chain C of the same structure or in the structure 1DDK (Fig. 1a).

Due to close proximity to the C2 tags in the N172C mutant, the largest PCSs were observed for Trp28 H ${}^{\varepsilon 1}$ but, in the absence of captopril, their exact magnitude appeared about 0.3 ppm smaller in the [ ${}^{15}$ N, ${}^{1}$ H]-HSQC (Fig. S5b) than the NOE-relayed [ ${}^{13}$ C, ${}^{1}$ H]-HSQC (Fig. 2b) spectrum. The centre of the localisation space of Trp28 H ${}^{\varepsilon 1}$ moved to a slightly more open L3 loop conformation when using the smaller PCS detected in the [ ${}^{15}$ N, ${}^{1}$ H]-HSQC spectrum of the N172C mutant labelled with the C2-Tb ${}^{3+}$ tag. The space still encompassed the coordinates observed in the structure 5EV6, limiting the significance of this difference in PCS.

None of the minor additional cross-peaks observed in any of the sample preparations could be attributed to alternative conformations of Trp28 either. In particular, the most extreme conformation observed in the crystal structure 1DDK (green in Fig. 1) predicts PCSs > 1 ppm for Trp28 H ${}^{\varepsilon 1}$ in the mutant N172C with C2 tags, but we observed no PCS of this magnitude for any of the unassigned peaks.

### 3.5 Defining the localisation space with one versus two lanthanoid ions in the same tag and at the same site

Unexpectedly, determining separate localisation spaces from the Tm ${}^{3+}$ and Tb ${}^{3+}$ data sets yielded more plausible results than when both data sets were used simultaneously. Careful inspection showed that the close alignment of the Δχ tensors of the Tm ${}^{3+}$ and Tb ${}^{3+}$ data resulted in particularly shallow intersection angles of the respective PCS isosurfaces. In calculating the localisation space of Trp28, the PCS isosurfaces arising from the N172C mutant carried by far the greatest weight as this site is closer to residue 28 than sites 53 and 204. Therefore, the Tm ${}^{3+}$ and Tb ${}^{3+}$ data from the N172C mutant dominated the PCS RMSD calculation and the intersection between the associated isosurfaces pulled the final localisation space to a structurally implausible location, which was unstable with respect to small perturbations in Δχ -tensor orientations associated with the tensors at site 172. In contrast, considering the Tm ${}^{3+}$ and Tb ${}^{3+}$ data sets separately allowed the localisation spaces to be determined by the intersections with PCS isosurfaces from the other sites. The resulting localisation spaces consistently were compatible with crystal structures.

### 3.6 L3 loop conformation in the presence of L-captopril

Figure 6 shows that, within the uncertainty of the experiments, the localisation space of the indole side chain of Trp28 is invariant with respect to the presence or absence of captopril. Conservation of the L3 loop conformation with and without inhibitor is supported by the close similarity in all the PCSs observed for Trp28 in the NOE-relayed [ ${}^{13}$ C, ${}^{1}$ H]-HSQC spectra (Figs. 2 and 3). In the [ ${}^{1}$ H, ${}^{15}$ N]-HSQC spectra of the mutant N172C with a C2 tag, however, the PCSs observed for Trp28 H ${}^{\varepsilon 1}$ appeared somewhat smaller without than with captopril (Fig. S5b). As the PCSs of backbone amides were very similar in the absence and presence of the inhibitor (Fig. S12), this difference in PCS suggests a change in L3 loop conformation, contradicting the observations made with the selectively ${}^{13}$ C-labelled samples. As discussed above, using the smaller PCS of Trp28 H ${}^{\varepsilon 1}$ did not sufficiently change its localisation space in the free protein to render it incompatible with the coordinates of the structure 5EV6. Therefore, as far as the data of the ${}^{15}$ N-labelled samples indicate a conformational change in the L3 loop between the free and bound states, it is small. We attribute the differences in PCSs observed between the selectively ${}^{13}$ C-labelled and uniformly ${}^{15}$ N-labelled samples to differences in sample preparation of unknown origin, which are also reflected by different numbers of weak unassigned cross-peaks (Figs. 2, 3, S5 and S6).

The cross-peak intensities of the Trp28 side-chain resonances are relatively weak compared with those of the other tryptophan side chains, suggesting that Trp28 is subject to dynamics that broaden its resonances. Its cross-peaks appeared slightly weaker in the presence than in the absence of inhibitor (Figs. 2 and 3), suggesting a change in dynamics caused by the inhibitor binding. Previous NMR studies of metallo- β -lactamases reported faster $R_{2}{(}^{15}$ N) relaxation rates of the L3-loop tryptophan side chain in the presence than in the absence of an inhibitor, which was attributed to dampened dynamics (Huntley et al., 2000; Softley et al., 2020). In the presence of dynamics, the localisation spaces determined in the present work must be considered averages that do not report on the amplitude or direction of motions.

### 3.7 Localisation spaces of tryptophan side chains other than Trp28

As the tagging sites had been designed to analyse the conformation of the L3 loop, they were positioned at similar distances from the L3 loop and were therefore not optimal for determining localisation spaces of the other tryptophan residues. Nonetheless, clear differences were observed in the PCSs of the H ${}^{\xi 2}$ and H ${}^{\varepsilon 1}$ atoms (Fig. 2), allowing the separation of the respective localisation spaces, which also proved to be in excellent agreement with the conformations of the side-chain indoles of Trp62, Trp124 and Trp147 as found in the crystal structure (Fig. S15), whereas the data were insufficient to determine the side-chain conformation of Trp176.

## 4 Discussion

The L3 loop of metallo- β -lactamases is known to be flexible and, in the specific case of IMP-1, significantly assists in substrate binding and enzymatic activity (Moali et al., 2003). As the substrate is sandwiched between the di-zinc site and the L3 loop, it is tempting to think that the loop opens up for substrate binding and product release, while it may be closed during the enzymatic reaction to hold the substrate and reaction intermediate in place. In contrast, some of the conformations observed in crystal structures of IMP-1 obtained in the presence and absence of the inhibitor L-captopril revealed the loop in almost identical conformations (Brem et al., 2016). This observation is inconclusive, however, as the L3 loop forms more extensive intermolecular contacts with neighbouring protein molecules in the crystal lattice than intramolecular contacts. In addition, other crystal structures observed the loop to move by almost 3 Å in response to a different inhibitor (Concha et al., 2000). This prompted us to probe its actual location in the absence of crystal packing forces in solution, a task which is difficult to tackle by traditional NMR spectroscopic methods that rely on short-range NOEs.

Our results show that by furnishing IMP-1 with paramagnetic lanthanoid tags, the coordinates of the indole side chain of Trp28, which is a key residue near the tip of the loop, can be determined with remarkable accuracy even in the free protein, where the available crystal structures position the L3 loop in a conformation without any direct contacts with the core of the protein. Indeed, the localisation space identified by the NMR data of the free protein proved to be sufficiently well defined to discriminate between different crystal structures of IMP-1 as well as between different chains in the same asymmetric crystal unit. For example, the side-chain orientation of Trp28 observed in [Fe ${}^{3+}$ ,Zn ${}^{2+}$ ]-IMP-1 (4UAM; Carruthers et al., 2014) proved to be in poor agreement with the PCS data, whereas the data were in full agreement with chain A in the structure 5EV6 of [Zn ${}^{2+}$ ,Zn ${}^{2+}$ ]-IMP-1 without an inhibitor (Hinchliffe et al., 2016) and chain A in the structure 4C1F with bound L-captopril (Brem et al., 2016). This highlights the outstanding capacity of PCSs to assess small conformational differences.

The approach of using PCSs for local structure determination is particularly appealing in the case of difficult proteins such as IMP-1, where the sequence-specific NMR resonance assignments are incomplete due to line broadening attributable to motions in the µ s–ms time range and additional signals are observed that either stem from protein degradation, misfolding or alternative conformations in slow exchange with the main structure. Notably, all information required to establish the Δχ tensors could be obtained from resolved cross-peaks observed in sensitive [ ${}^{15}$ N, ${}^{1}$ H]-HSQC spectra. Similarly, the localisation information of the tryptophan side chains could be obtained from sensitive ${}^{13}$ C- ${}^{1}$ H and ${}^{15}$ N- ${}^{1}$ H correlation spectra. Positioning the lanthanoid tags relatively far from the substrate binding site avoided direct interference with the binding loop structure.

In the face of additional signals from minor species, site-selective ${}^{13}$ C labelling of the tryptophan side chains was particularly helpful for simplifying the [ ${}^{13}$ C, ${}^{1}$ H]-HSQC spectra. Gratifyingly, this could be achieved by providing suitably labelled indole without having to synthesise the full amino acid (Maleckis et al., 2021).

It has been pointed out previously that the accuracy with which localisation spaces can be determined is best when PCS isosurfaces intersect in an orthogonal manner (Pintacuda et al., 2006; Lescanne et al., 2018; Zimmermann et al., 2019). In the present work, we found that, counterintuitively, the provision of additional data can considerably degrade the accuracy of the localisation space. This effect arises when PCS isosurfaces intersect at a shallow angle, as the location of these intersections becomes very sensitive with regard to small errors in the relative orientations of the underpinning Δχ tensors. Shallow intersection angles of PCS isosurfaces are common when two PCS data sets are from tags and tagging sites that differ only in the identity of the paramagnetic metal ion in the tag. This situation commonly generates Δχ tensors of different magnitude and sign but closely similar orientation (Bertini et al., 2001; Su et al., 2008; Keizers et al., 2008; Man et al., 2010; Graham et al., 2011; Joss et al., 2018; Zimmermann et al., 2019). Therefore, while the use of Tm ${}^{3+}$ and Tb ${}^{3+}$ tags is helpful for assigning the cross-peaks in the paramagnetic state, more robust results are obtained by using only one of these data sets for calculating the localisation space. Good localisation spaces were thus obtained by using only PCSs measured for Tb ${}^{3+}$ tags (Fig. 6) or only PCSs measured for Tm ${}^{3+}$ tags (Fig. S13). In contrast, however, very different tags attached at the same site, such as the C2 and C12 tags installed in the mutant N172C, produced independent Δχ -tensor orientations and therefore contributed positively to localising the Trp28 H ${}^{\varepsilon 1}$ atom.

In principle it is inappropriate to explain a set of PCSs by a single Δχ tensor if they are generated by a lanthanoid tag attached via a flexible linker, which positions the lanthanide ions at variable coordinates relative to the protein. In this situation, fitting a single Δχ tensor amounts to an approximation. The effective Δχ tensors obtained in this way, however, can fulfill the PCSs remarkably well (Shishmarev and Otting, 2013), as illustrated by the low Q factors obtained in this work (Fig. 4), and the localisation spaces obtained for the tryptophan side chains are correspondingly well defined.

The accuracy with which localisation spaces can be determined further depends on the accuracy with which PCSs can be measured (which critically depends on the reproducibility of the sample conditions between the paramagnetic and diamagnetic states), the accuracy of the protein structure used to fit the Δχ tensors and the angle with which PCS isosurfaces of different tensors intersect. To take into account the uncertainties associated with the PCS isosurfaces, it is useful to think of each of them individually as a shell of a certain thickness (rather than a surface) that represents a compatible localisation space. Two shells of a given thickness share a smaller common space if they intersect orthogonally than if they intersect at a shallow angle.

The present work employed ${}^{1}$ H PCSs only, although PCSs were also observed in the indirect dimensions of the [ ${}^{13}$ C, ${}^{1}$ H]-HSQC and [ ${}^{15}$ N, ${}^{1}$ H]-HSQC spectra. We made this choice because the paramagnetic tags give rise to weak molecular alignments in the magnetic field, which result in residual anisotropic chemical shifts (RACSs). The effect is unimportant for ${}^{1}$ H spins but significant for nuclear spins with large chemical shift anisotropy (CSA) tensors such as backbone nitrogens and aromatic carbons. Correcting for the RACS effect is possible with prior knowledge of the CSA tensors and bond orientations (John et al., 2005). We chose not to measure PCSs of the heteronuclear spins in favour of improving sensitivity by accepting a lower spectral resolution in the indirect dimensions.

Finally, the C12 tag was designed specifically with the intent to produce a more rigid tether to the protein than the C2 tag, but this did not result in larger Δχ tensors (Table S7), and the NMR spectra of IMP-1 N172C displayed more heterogeneity with the C12 than C2 tag, suggesting that the shorter and more rigid tether combined with the fairly high molecular weight of the cyclen–lanthanoid complex may have perturbed the protein structure to some degree.

## 5 Conclusion

The current work illustrates how Δχ tensors from paramagnetic lanthanoid ion tags installed at three different sites of the protein can be used to probe the conformation of a selected site in solution in unprecedented detail, provided the structure of most of the protein is known with high accuracy to allow fitting of effective Δχ tensors of high predictive value. Importantly, however, the method is easily compromised if two PCS isosurfaces intersect at a shallow angle as, in this situation, inaccuracies in Δχ -tensor determinations have an outsized effect on positioning the localisation spaces defined by the PCSs. Therefore, improved results were obtained by not combining data from different metal ions bound to otherwise identical tags and tagging sites. In the present work, simplifying the NMR spectrum of tryptophan residues by site-selective isotope labelling proved to be of great value for sufficiently improving the spectral resolution to allow assignment of the labelled resonances solely from PCSs and PREs. The strategy opens a path to detailed structural investigations of proteins of limited stability like IMP-1, for which complete assignments of the NMR spectrum are difficult to obtain.

## Supplement

10.5194/mr-3-1-2022-supplement The supplement related to this article is available online at: https://doi.org/10.5194/mr-3-1-2022-supplement.

## Data Availability

NMR spectra and pulse programs are available at https://doi.org/10.5281/zenodo.5518294 (Orton et al., 2021). The script for calculating localisation spaces is available at https://doi.org/10.5281/zenodo.3594568 (Orton, 2019) and from the GitHub site of Paramagpy.

## References

[bib1.bib1] Arakawa Y, Murakami M, Suzuki K, Ito H, Wacharotayankun R, Ohsuka S, Kato N, Ohta M (1995). A novel integron-like element carrying the metallo-
β
-lactamase gene *bla*

${}_{\text{IMP}}$. Antimicrob Agents Chemother.

[bib1.bib2] Bertini I, Janik MBL, Lee YM, Luchinat C, Rosato A (2001). Magnetic susceptibility tensor anisotropies for a lanthanide ion series in a fixed protein matrix. J Am Chem Soc.

[bib1.bib3] Brem J, van Berkel SS, Zollman D, Lee SY, Gileadi O, McHugh PJ, Walsh TR, McDonough MA, Schofield CJ (2016). Structural basis of metallo-
β
-lactamase inhibition by captopril stereoisomers. Antimicrob Agents Chemother.

[bib1.bib4] Brewer KD, Bacaj T, Cavalli A, Camilloni C, Swarbrick JD, Liu J, Zhou A, Zhou P, Barlow N, Xu J, Seven AB, Prinslow EA, Voleti R, Häussinger D, Bonvin AMJJ, Tomchick DR, Vendruscolo M, Graham B, Südhof TC, Rizo J (2015). Dynamic binding mode of a synaptotagmin-1-SNARE complex in solution. Nat Struct Mol Biol.

[bib1.bib5] Bush K (2010). Alarming 
β
-lactamase-mediated resistance in multidrug-resistant *Enterobacteriaceae*. Curr Opin Microbiol.

[bib1.bib6] Bush K (2013). Proliferation and significance of clinically relevant 
β
-lactamases. Ann N Y Acad Sci.

[bib1.bib7] Carruthers TJ, Carr PD, Loh C-T, Jackson CJ, Otting G (2014). Fe
${}^{3+}$
 located in the dinuclear metallo-
β
-lactamase IMP-1 by pseudocontact shifts. Angew Chemie Int Ed.

[bib1.bib8] Chen W.N, Nitsche C, Pilla KB, Graham B, Huber T, Klein CD, Otting G (2016). Sensitive NMR approach for determining the binding mode of tightly binding ligand molecules to protein targets. J Am Chem Soc.

[bib1.bib9] Concha NO, Rasmussen BA, Bush K, Herzberg O (1996). Crystal structure of the wide-spectrum binuclear zinc 
β
-lactamase from *Bacteroides fragilis*. Structure.

[bib1.bib10] Concha NO, Janson CA, Rowling P, Pearson S, Cheever CA, Clarke BP, Lewis C, Galleni M, Frere J-M, Payne DJ, Bateson JH, Abdel-Meguid SS (2000). Crystal Structure of the IMP-1 metallo-
β
-lactamase from *Pseudomonas aeruginosa* and its complex with a mercaptocarboxylate inhibitor: binding determinants of a potent, broad-spectrum inhibitor. Biochemistry.

[bib1.bib11] Crick DJ, Wang JX, Graham B, Swarbrick JD, Mott HR, Nietlispach D (2015). Integral membrane protein structure determination using pseudocontact shifts. J Biomol NMR.

[bib1.bib12] de la Cruz L, Nguyen THD, Ozawa K, Shin J, Graham B, Huber T, Otting G (2011). Binding of low-molecular weight inhibitors promotes large conformational changes in the dengue virus NS2B-NS3 protease:
fold analysis by pseudocontact shifts. J Am Chem Soc.

[bib1.bib13] Galleni M, Lamotte-Brasseur J, Rossolini GM, Spencer J, Dideberg O, Frère J-M, The Metallo-β-Lactamase Working Group (2001). Standard numbering scheme for class B 
β
-lactamases. Antimicrob Agents Chemother.

[bib1.bib14] Gianquinto E, Tondi D, D'Arrigo G, Lazzarato L, Spyrakis F (2020). Can we exploit 
β
-lactamases intrinsic dynamics for designing more effective inhibitors?. Antibiotics.

[bib1.bib15] González MM, Abriata LA, Tomatis PE, Vila AJ (2016). Optimization of conformational dynamics in an epistatic evolutionary trajectory. Mol Biol Evol.

[bib1.bib16] Graham B, Loh CT, Swarbrick JD, Ung P, Shin J, Yagi H, Jia X, Chhabra S, Pintacuda G, Huber T, Otting G (2011). A DOTA-amide lanthanide tag for reliable generation of pseudocontact shifts in protein NMR spectra. Bioconjugate Chem.

[bib1.bib17] Guan JY, Keizers PHJ, Liu WM, Löhr F, Skinner SP, Heeneman EA, Schwalbe H, Ubbink M, Siegal G (2013). Small-molecule binding sites on proteins established by paramagnetic NMR spectroscopy. J Am Chem Soc.

[bib1.bib18] Herath ID, Breen C, Hewitt S.H, Berki TR, Kassir AF, Dodson C, Judd M, Jabar S, Cox N, Otting G, Butler SJ (2021). A chiral lanthanide tag for stable and rigid attachment to single cysteine residues in proteins for NMR, EPR and time-resolved luminescence studies. Chem Eur J.

[bib1.bib19] Hinchliffe P, González MM, Mojica MF, González JM, Castillo V, Saiz C, Kosmopoulou M, Tooke CL, Llarrull LI, Mahler G, Bonomo RA (2016). Cross-class metallo-
β
-lactamase inhibition by bisthiazolidines reveals multiple binding modes. Proc Nat Acad Sci.

[bib1.bib20] Hinchliffe P, Tanner CA, Krismanich AP, Labbé G, Goodfellow VJ, Marrone L, Desoky AY, Calvopiña K, Whittle EE, Zeng F, Avison MB (2018). Structural and kinetic studies of the potent inhibition of metallo-
β
-lactamases by 6-phosphonomethylpyridine-2-carboxylates. Biochemistry.

[bib1.bib21] Hiraiwa Y, Saito J, Watanabe T, Yamada M, Morinaka A, Fukushima T, Kudo T (2014). X-ray crystallographic analysis of IMP-1 metallo-
β
-lactamase complexed with a 3-aminophthalic acid derivative, structure-based drug design, and synthesis of 3, 6-disubstituted phthalic acid derivative inhibitors. Bioorg Med Chem Lett.

[bib1.bib22] Huntley JJA, Scrofani SDB, Osborne MJ, Wright PE, Dyson HJ (2000). Dynamics of the metallo-
β
-lactamase from *Bacteroides fragilis* in the presence and absence of a tight-binding inhibitor. Biochemistry.

[bib1.bib23] Huntley JJA, Fast W, Benkovic SJ, Wright PE, Dyson HJ (2003). Role of a solvent-exposed tryptophan in the recognition and binding of antibiotic substrates for a metallo-
β
-lactamase. Protein Sci.

[bib1.bib24] Ito H, Arakawa Y, Ohsuka S, Wachorotayankun R, Kato N, Ohta M (1995). Plasmid-mediated dissemination of the metallo-
β
-lactamase gene *bla*

${}_{\text{IMP}}$
 among clinically isolated strains of *Serratia marcescens*. Antimicrob Agents Chemother.

[bib1.bib25] John M, Park AY, Pintacuda G, Dixon NE, Otting G (2005). Weak alignment of paramagnetic proteins warrants correction for residual CSA effects in measurements of pseudocontact shifts. J Am Chem Soc.

[bib1.bib26] Joss D, Walliser RM, Zimmermann K, Häussinger D (2018). Conformationally locked lanthanide chelating tags for convenient pseudocontact shift protein nuclear magnetic resonance spectroscopy. J Biomol NMR.

[bib1.bib27] Keizers PHJ, Saragliadis A, Hiruma Y, Overhand M, Ubbink M (2008). Design, synthesis, and evaluation of a lanthanide chelating protein probe: CLaNP-5 yields predictable paramagnetic effects independent of environment. J Am Chem Soc.

[bib1.bib28] Keizers PHJ, Mersinli B, Reinle W, Donauer J, Hiruma Y, Hannemann F, Overhand M, Bernhardt R, Ubbink M (2010). A solution model of the complex formed by adrenodoxin and adrenodoxin reductase determined by paramagnetic NMR spectroscopy. Biochemistry.

[bib1.bib29] Kobashigawa Y, Saio T, Ushio M, Sekiguchi M, Yokochi M, Ogura K, Inagaki F (2012). Convenient method for resolving degeneracies due to symmetry of the magnetic susceptibility tensor and its application to pseudo contact shift-based protein-protein complex structure determination. J Biomol NMR.

[bib1.bib30] Laraki N, Galleni M, Thamm I, Riccio ML, Amicosante G, Frère J-M, Rossolini GM (1999). Structure of In101, a *bla*

${}_{\text{IMP}}$
-containing *Pseudomonas aeruginosa* integron phyletically related to In5, which carries an unusual array of gene cassettes. Antimicrob Agents Chemother.

[bib1.bib31] Laraki N, Franceschini N, Rossolini GM, Santucci P, Meunier C, De Pauw E, Amicosante G, Frère J-M, Galleni M (1999). Biochemical characterization of the *Pseudomonas aeruginosa* 101/1477 metallo-
β
-lactamase IMP-1 produced by *Escherichia coli*. Antimicrob Agents Chemother.

[bib1.bib32] Lescanne M, Ahuja P, Blok A, Timmer M, Akerud T, Ubbink M (2018). Methyl group reorientation under ligand binding probed by pseudocontact shifts. J Biomol NMR.

[bib1.bib33] Linciano P, Cendron L, Gianquinto E, Spyrakis F, Tondi D (2019). Ten years with New Delhi metallo-
β
-lactamase-1 (NDM-1): from structural insights to inhibitor design. ACS Infect Dis 5.

[bib1.bib34] Maleckis A, Herath ID, Otting G (2021). Synthesis of 
${}^{13}$
C/
${}^{19}$
F/
${}^{2}$
H labeled indoles for use as tryptophan precursors for protein NMR spectroscopy. Org Biomol Chem.

[bib1.bib35] Man B, Su X-C, Liang H, Simonsen S, Huber T, Messerle BA, Otting G (2010). 3-Mercapto-2,6-pyridinedicarboxylic acid, a small lanthanide-binding tag for protein studies by NMR spectroscopy. Chem Eur J.

[bib1.bib36] Markley JL, Bax A, Arata Y, Hilbers CW, Kaptein R, Sykes BD, Wright PE, Wüthrich K (1998). Recommendations for the presentation of NMR structures of proteins and nucleic acids –
IUPAC-IUBMB-IUPAB Inter-Union Task Group on the Standardization of Data Bases of Protein and Nucleic Acid Structures Determined by NMR Spectroscopy. J Biomol NMR.

[bib1.bib37] Meissner A, Duus JØ, Sørensen OW (1997). Spin-state-selective excitation. Application for E.COSY-type measurement of 
$J_{\text{HH}}$
 coupling constants. J Magn Reson.

[bib1.bib38] Moali C, Anne C, Lamotte-Brasseur J, Groslambert S, Devreese B, Van Beeumen J, Galleni M, Frère JM (2003). Analysis of the importance of the metallo-
β
-lactamase active site loop in substrate binding and catalysis. Chem Biol.

[bib1.bib39] Orton H, Otting G, Herath I (2021). Zenodo.

[bib1.bib40] Orton HW (2019). Zenodo.

[bib1.bib41] Orton HW, Huber T, Otting G (2020). Paramagpy: software for fitting magnetic susceptibility tensors using paramagnetic effects measured in NMR spectra. Magn Reson.

[bib1.bib42] Palacios AR, Mojica MF, Giannini E, Taracila MA, Bethel CR, Alzari PM, Otero LH, Klinke S, Llarrull LI, Bonomo RA, Vila AJ (2019). The reaction mechanism of metallo-
β
-lactamases is tuned by the conformation of an active-site mobile loop. Antimicrob Agents Chemother.

[bib1.bib43] Payne DJ, Hueso-Rodriguez JA, Boyd H, Concha NO, Janson CA, Gilpin M, Bateson JH, Cheever C, Niconovich NL, Pearson S, Rittenhouse S, Tew D, Díez E, Pérez P, de la Fuente J, Rees M, Rivera-Sagredo A (2002). Identification of a series of tricyclic natural products as potent broad-spectrum inhibitors of metallo-
β
-lactamases. Antimicrob Agents Chemother.

[bib1.bib44] Pearce BJG, Jabar S, Loh CT, Szabo M, Graham B, Otting G (2017). Structure restraints from heteronuclear pseudocontact shifts generated by lanthanide tags at two different sites. J Biomol NMR.

[bib1.bib45] Pilla KB, Otting G, Huber T (2017). Protein structure determination by assembling super-secondary structure motifs using pseudocontact shifts. Structure.

[bib1.bib46] Pintacuda G, Park AY, Keniry MA, Dixon NE, Otting G (2006). Lanthanide labeling offers fast NMR approach to 3D structure determinations of protein-protein complexes. J Am Chem Soc.

[bib1.bib47] Qi R, Otting G (2019). Mutant T4 DNA polymerase for easy cloning and mutagenesis. PLOS One.

[bib1.bib48] Rossi M-A, Martinez V, Hinchliffe P, Mojica MF, Castillo V, Moreno DM, Smith R, Spellberg B, Drusano GL, Banchio C, Bonomo RA, Spencer J, Vila AJ, Mahler G (2021). 2-Mercaptomethyl-thiazolidines use conserved aromatic–S interactions to achieve broad-range inhibition of metallo-
β
-lactamases. Chem Sci.

[bib1.bib49] Salimraj R, Hinchliffe P, Kosmopoulou M, Tyrrell JM, Brem J, van Berkel SS, Verma A, Owens RJ, McDonough MA, Walsh TR, Schofield CJ, Spencer J (2018). Crystal structures of VIM-1 complexes explain active site heterogeneity in VIM-class metallo-
β
-lactamases. FEBS J.

[bib1.bib50] Shishmarev D, Otting G (2013). How reliable are pseudocontact shifts induced in proteins and ligands by mobile paramagnetic metal tags? A modelling study. J Biomol NMR.

[bib1.bib51] Softley CA, Zak KM, Bostock MJ, Fino R, Zhou RX, Kolonko M, Mejdi-Nitiu R, Meyer H, Sattler M, Popowicz GM (2020). Structure and molecular recognition mechanism of IMP-13 metallo-
β
-lactamase. Antimicrob Agents Chemother.

[bib1.bib52] Su X-C, McAndrew K, Huber T, Otting G (2008). Lanthanide-binding peptides for NMR measurements of residual dipolar couplings and paramagnetic effects from multiple angles. J Am Chem Soc.

[bib1.bib53] Toney JH, Hammond GG, Fitzgerald PM, Sharma N, Balkovec JM, Rouen GP, Olson SH, Hammond ML, Greenlee ML, Gao YD (2001). Succinic acids as potent inhibitors of plasmid-borne IMP-1 metallo-
β
-lactamase. J Biol Chem.

[bib1.bib54] van Duin D, Kaye KS, Neuner EA, Bonomo RA (2013). Carbapenem-resistant Enterobacteriaceae: a review of treatment and outcomes. Diagn Microbiol Infect Dis.

[bib1.bib55] Wachino J, Kanechi R, Nishino E, Mochizuki M, Jin W, Kimura K, Kurosaki H, Arakawa Y (2019). 4-Amino-2-sulfanylbenzoic acid as a potent subclass B3 metallo-
β
-lactamase-specific inhibitor applicable for distinguishing metallo-
β
-lactamase subclasses. Antimicrob Agents Chemother.

[bib1.bib56] Watanabe M, Iyobe S, Inoue M, Mitsuhashi S (1991). Transferable imipenem resistance in *Pseudomonas aeruginosa*. Antimicrob Agents Chemother.

[bib1.bib57] Wu PSC, Ozawa K, Lim SP, Vasudevan S, Dixon NE, Otting G (2007). Cell-free transcription/translation from PCR amplified DNA for high-throughput NMR studies. Angew Chemie Int Ed.

[bib1.bib58] Yagi H, Pilla KB, Maleckis A, Graham B, Huber T, Otting G (2013). Three-dimensional protein fold determination from backbone amide pseudocontact shifts generated by lanthanide tags at multiple sites. Structure.

[bib1.bib59] Yamaguchi Y, Matsueda S, Matsunaga K, Takashio N, Toma-Fukai S, Yamagata Y, Shibata N, Wachino J, Shibayama K, Arakawa Y, Kurosaki H (2015). Crystal structure of IMP-2 metallo-
β
-lactamase from *Acinetobacter* spp.: comparison of active-site loop structures between IMP-1 and IMP-2. Biol Pharm Bull.

[bib1.bib60] Zimmermann K, Joss D, Müntener T, Nogueira ES, Schäfer M, Knörr L, Monnard FW, Häussinger D (2019). Localization of ligands within human carbonic anhydrase II using 
${}^{19}$
F pseudocontact shift analysis. Chem Sci.

