# Supplementary material for: Localising individual atoms of tryptophan side chains in the metallo-β-lactamase IMP-1 by pseudocontact shifts from paramagnetic lanthanoid tags at multiple sites
Source: Magn Reson (Gott). 2022 Jan 4;3(1):1–13. doi: 10.5194/mr-3-1-2022 (PMC10583275; doi:10.5194/mr-3-1-2022)
Supplement: The supplement related to this article is available online at: https://doi.org/10.5194/mr-3-1-2022-supplement. [file mr-3-1-supplement.pdf]

Supplement of Magn. Reson., 3, 1–13, 2022
https://doi.org/10.5194/mr-3-1-2022-supplement
© Author(s) 2022. CC BY 4.0 License.

Open Access
MAGNETIC RESONANCE

Supplement of

Localising individual atoms of tryptophan side chains in the metallo-β-lactamase IMP-1 by pseudocontact shifts from paramagnetic lanthanoid tags at multiple sites

Henry W. Orton et al.

Correspondence to: Gottfried Otting (gottfried.otting@anu.edu.au)

The copyright of individual parts of the supplement might differ from the article licence.

Supporting Information

Table of Contents

Protein mass spectrometry

Figure S1. Chemical structures of the C2 and C12 tags

Figure S2. Mass spectra of cysteine mutants of IMP-1 showing the incorporation of $^{13}C^{\zeta}$ labelled deuterated indoles and ligation yields with the C2-Y^{3+} tag

Figure S3. Superimpositions of [^{15}N,^{1}H]-HSQC spectra of IMP-1 tagged with C2-Ln^{3+} tags

Figure S4. Superimpositions of [^{15}N,^{1}H]-HSQC spectra of IMP-1 tagged with C2-Ln^{3+} tags and in the presence of captopril

Figure S5. Spectral region of the [^{15}N,^{1}H]-HSQC spectra of Figures S3 and S4 showing the indole $N^{\varepsilon 1}$–$H^{\varepsilon 1}$ cross-peaks

Figure S6. Spectral region of the [^{15}N,^{1}H]-HSQC spectrum of wild-type IMP-1 selectively labelled with ^{15}N-tryptophan

Figure S7. Superimpositions of [^{15}N,^{1}H]-HSQC spectra recorded of IMP-1 N172C ligated with the C12 tag, in the absence and presence of captopril

Figure S8. Spectral region of the [^{15}N,^{1}H]-HSQC spectra of Figure S4 showing the tryptophan $N^{\zeta 2}$–$H^{\zeta 2}$ cross-peaks

Figure S9. Pulse sequence of [^{13}C,^{1}H]-HSQC with S^3E filter designed to select the low-field TROSY component in the ^{13}C dimension

Figure S10. Pulse sequence of [^{13}C,^{1}H]-HSQC with NOE relay

Figure S11. *Q* factors of $\Delta\chi$-tensor fits to ^{1}H PCSs measured of backbone amides for different PDB structures

Figure S12. Correlations of ^{1}H PCSs measured of backbone amides in the presence versus the absence of captopril

Figure S13. PCS isosurfaces obtained with three C2-Tb^{3+} tags plotted for the Trp28 $H^{\varepsilon 1}$ atom

Figure S14. Localisation spaces of the Trp28 $H^{\zeta 2}$ and $H^{\varepsilon 1}$ atoms defined by PCSs obtained with Tm^{3+} tags

Figure S15. Localisation spaces of the $H^{\zeta 2}$ and $H^{\varepsilon 1}$ atoms of Trp62, Trp88, Trp124 and Trp147

Table S1. Pseudocontact shifts of the backbone amide protons of IMP-1 cysteine mutants ligated with lanthanoid tags

Table S2. Pseudocontact shifts of the backbone amide protons of IMP-1 cysteine mutants ligated with lanthanoid tags and in the presence of captopril

Table S3. Pseudocontact shifts of the $H^{\zeta 2}$ proton of the tryptophan residues in the IMP-1 cysteine mutants ligated with C2-Tb^{3+} or C2-Tm^{3+} tags

Table S4. Pseudocontact shifts of the tryptophan $H^{\varepsilon 1}$ proton in the IMP-1 cysteine mutants ligated with C2 and C12 lanthanoid tags

Table S5. Pseudocontact shifts of the tryptophan $H^{\zeta 2}$ protons in the IMP-1 cysteine mutants ligated with C2 and C12 lanthanoid tags and in the presence of captopril

Table S6. Pseudocontact shifts of the tryptophan $H^{\varepsilon 1}$ protons in the IMP-1 cysteine mutants ligated with lanthanoid tags in the presence of captopril

Table S7. $\Delta\chi$ tensor parameters fitted to different IMP-1 structures using ^{1}H PCSs of backbone amides

Table S8. Uncertainty ranges associated with the $\Delta\chi$-tensor parameters of Table S7

References

Protein mass spectrometry

Intact protein analysis was carried out on an Orbitrap Elite Hybrid Ion Trap–Orbitrap mass spectrometer connected to an UltiMate 3000 UHPLC (Thermo Scientific, USA). 0.1% formic acid and an acetonitrile gradient (10–85 %) were used to inject the samples into the mass analyzer via an Agilent ZORBAX SB-C3 Rapid Resolution HT Threaded Column. Data were collected in positive ion mode using an electrospray ionization (ESI) source. The Xtract function in the Qual Browser software tool of the program Xcalibur 3.0.63 was used to deconvolute and determine the protein intact mass. (Thermo Fisher Scientific, USA).

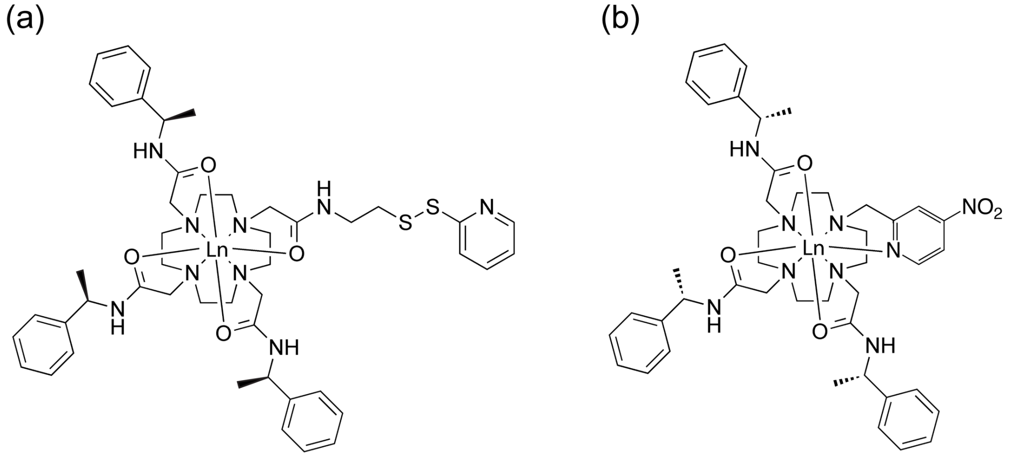

Figure S1. Chemical structures of the (a) C2 and (b) C12 lanthanoid tags. The activated disulfide bond in the C1 tag reacts with a cysteine thiol group with formation of a new disulfide bond. In the C12 tag, the nitro group acts as a leaving group in the reaction with a cysteine thiol, leading to the formation of a thioether.

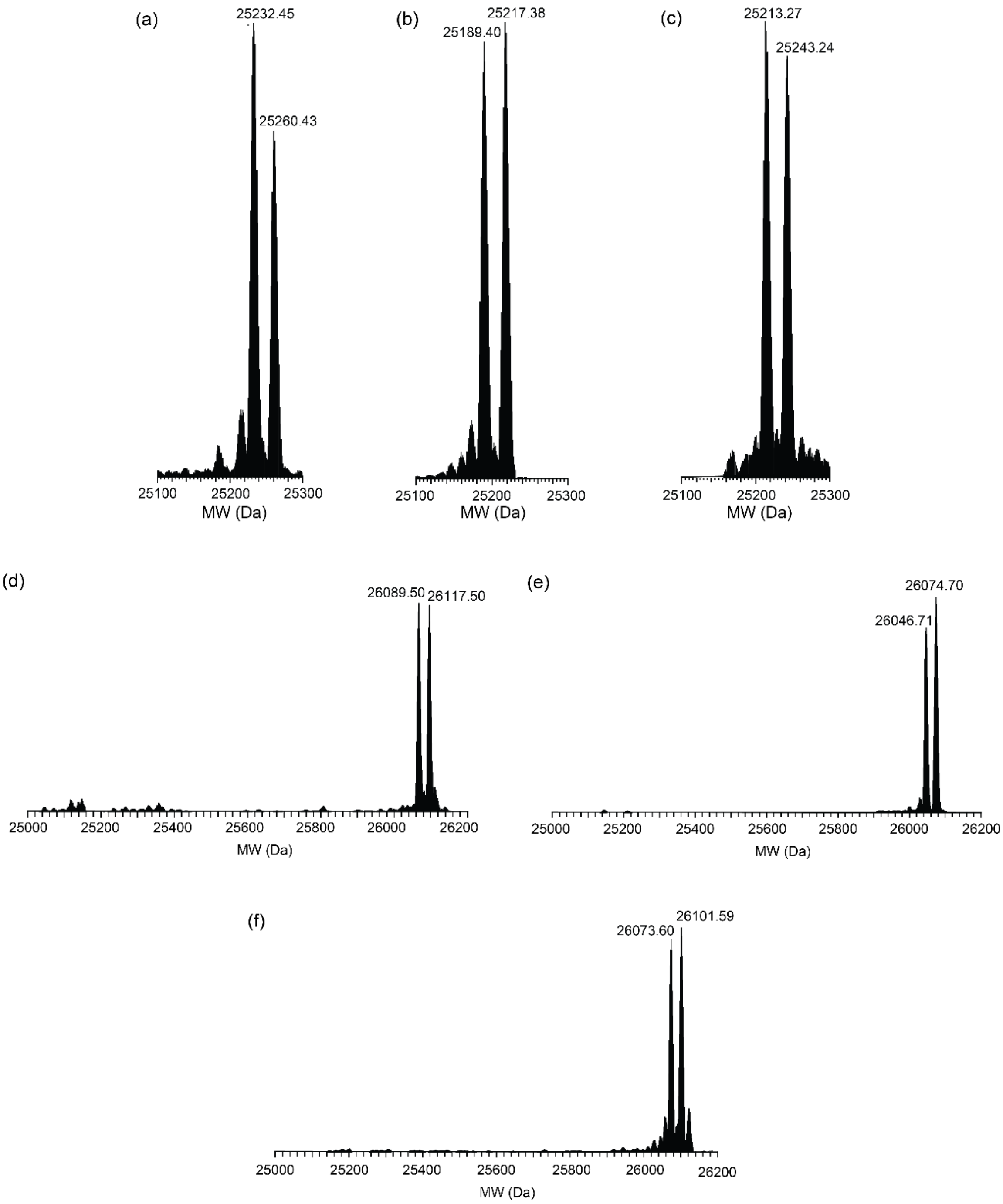

Figure S2. Mass spectra of cysteine mutants of IMP-1 showing the incorporation of $^{13}C^{\zeta2}$-labelled and deuterated indoles and ligation yields with the C2-Y^{3+} tag. The second major peak in each spectrum is attributed to N-terminal formylation, which adds 28 Da. The ligation with the tag increases the mass by 860 Da. (a) A53C mutant. Calculated mass for six labelled tryptophan residues: 25235 Da (5 Da less, if only five tryptophans are labelled). (b) N172C mutant. Calculated mass: 25192 Da, if all six tryptophan residues are labelled. (c) S204C mutant. Calculated mass: 25219 Da, if all six tryptophans are labelled. (d) Same as (a), but after tagging. (e) Same as (b) following tagging. (f) Same as (c) following tagging.

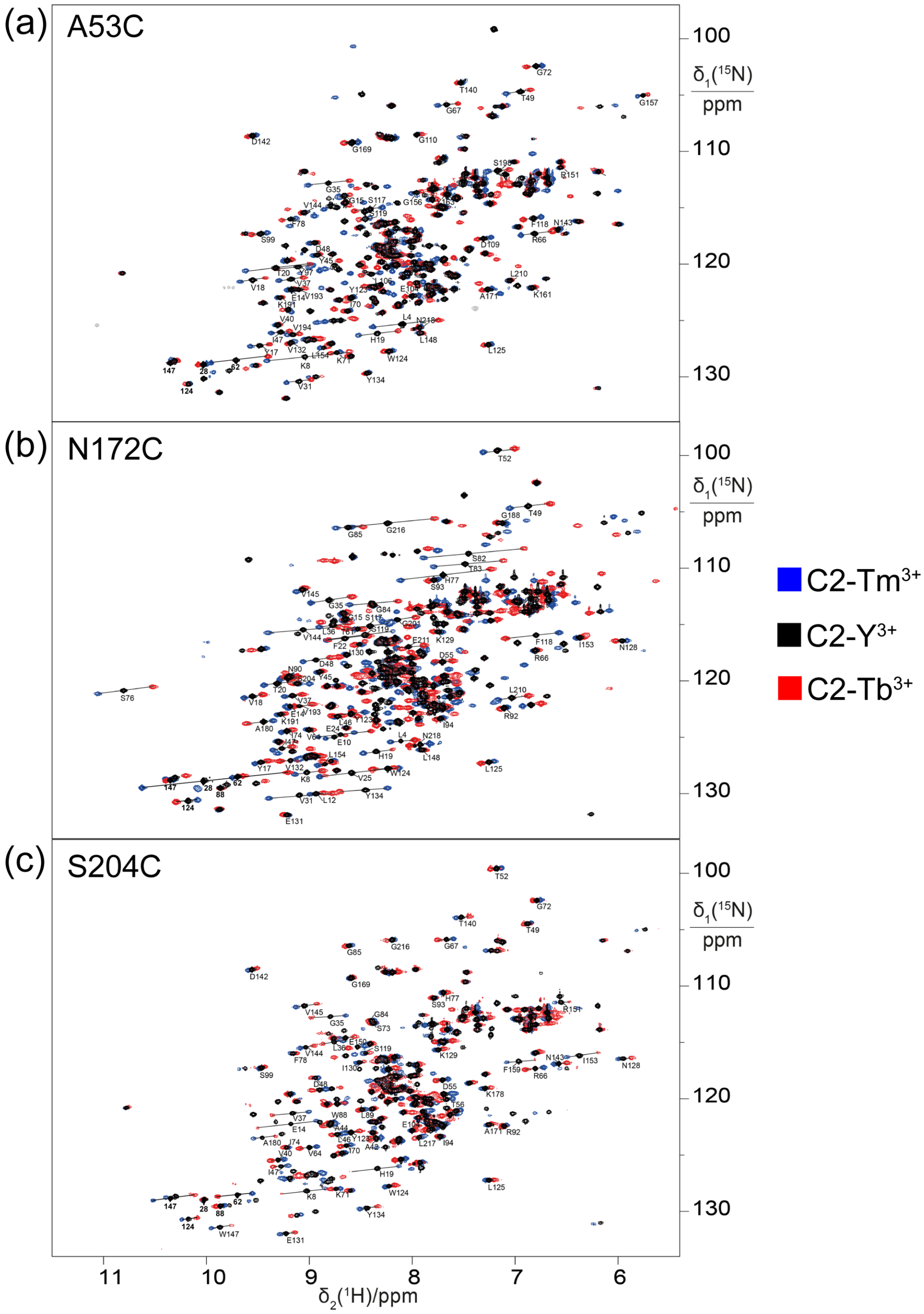

Figure S3. Superimpositions of [^{15}N,^{1}H]-HSQC spectra recorded of 0.6 mM solutions of uniformly ^{15}N-labelled IMP-1 tagged with C2-Ln^{3+} at three different sites. Spectra with diamagnetic tag (C2-Y^{3+}) are plotted in black and the corresponding spectra with paramagnetic tags are shown in red (C2-Tb^{3+}) and blue (C2-Tm^{3+}). All spectra were measured at 310 K in NMR buffer (20 mM MES, pH 6.5, 100 mM NaCl) on a Bruker 800 MHz NMR spectrometer. Some of the PCSs are indicated by lines connecting the peaks of paramagnetic and diamagnetic samples. Cross-peaks of tryptophan indole NH groups in the diamagnetic samples are labelled in bold with the residue number only. (a) Mutant A53C. (b) Mutant N172C. (c) Mutant S204C.

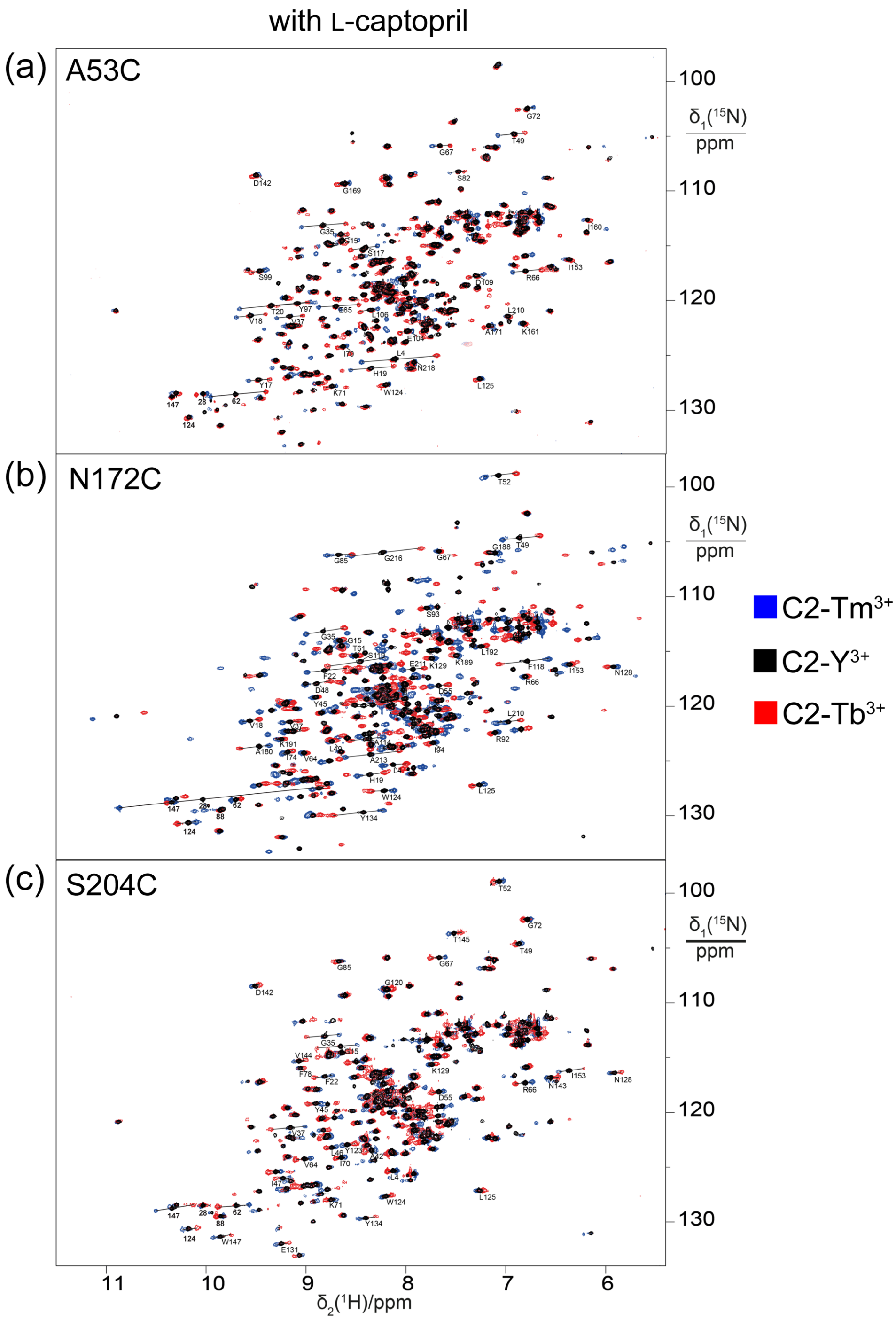

Figure S4. Superimpositions of [^{15}N,^{1}H]-HSQC spectra recorded of 0.6 mM solutions of uniformly ^{15}N-labelled IMP-1 tagged with C2-Ln^{3+} at three different sites in the presence 1.5-fold excess of captopril. All other parameters and annotations are the same as in Figure 1. (a) Mutant A53C. (b) Mutant N172C. (c) Mutant S204C.

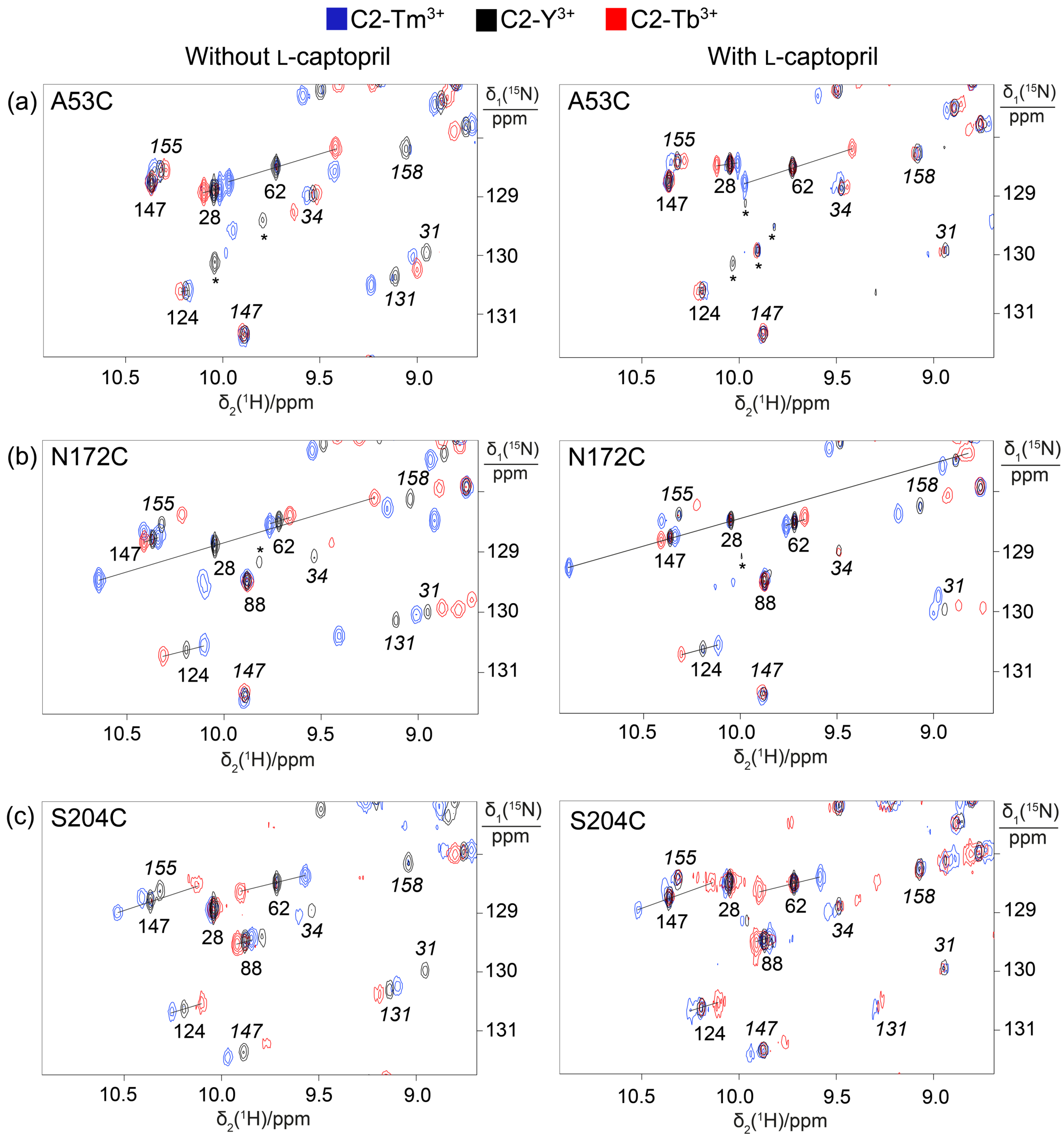

Figure S5. Spectral region of the [^{15}N,^{1}H]-HSQC spectra of Figures S3 and S4 showing the tryptophan $N^{\varepsilon1}$–$H^{\varepsilon1}$ cross-peaks without (left panels) and with (right panels) captopril. Cross-peaks of backbone amides are assigned in italics. Sidechain assignments of tryptophan residues are in regular font. (a) Mutant A53C. (b) Mutant N172C. (c) Mutant S204C. Tryptophan $N^{\varepsilon1}$–$H^{\varepsilon1}$ cross-peaks were assigned by comparison with the ^{1}H chemical shifts observed in a selectively ^{15}N-Trp-labelled sample (Fig. S6) and the PCSs observed in the NOE-relayed [^{13}C,^{1}H]-HSQC spectra of Figures 2 and 3. This comparison did not allow secure identification of the $N^{\varepsilon1}$–$H^{\varepsilon1}$ cross-peak of Trp176 in any of the [^{15}N,^{1}H]-HSQC spectra and the cross-peak of Trp88 could not be assigned for the mutant A53C (residue 53 is spatially close to Trp88). Stars mark cross-peaks in the samples prepared with C2-Y^{3+} tag, which could not be assigned.

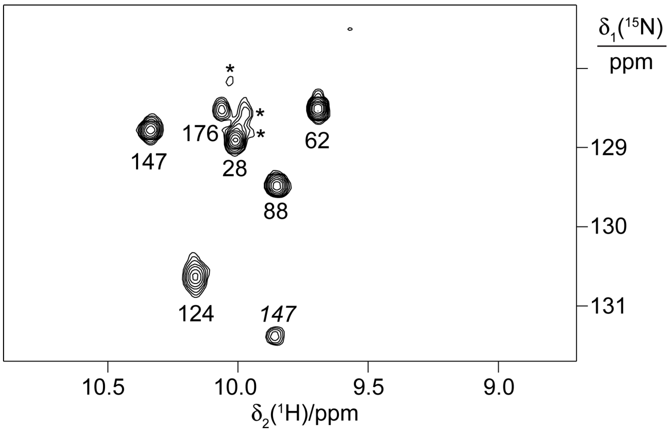

Figure S6. Selected spectral region of the [^{15}N,^{1}H]-HSQC spectrum of wild-type IMP-1 selectively labelled with ^{15}N-tryptophan (Carruthers 2014). The plot shows the assignment of the $N^{\varepsilon 1}$–$H^{\varepsilon 1}$ cross-peaks of the sidechains obtained in the present work. The assignment of Trp176 is tentative, as the cross-peak was observed in the NOE-relayed [^{13}C,^{1}H]-HSQC spectra of Fig. 2, but not in the [^{15}N,^{1}H]-HSQC spectra of Fig. S5. The cross-peak of the backbone amide of Trp147 is labelled in italics. Stars identify weak cross-peaks arising from sample heterogeneity. They are of unknown origin and were not reproduced between different sample preparations.

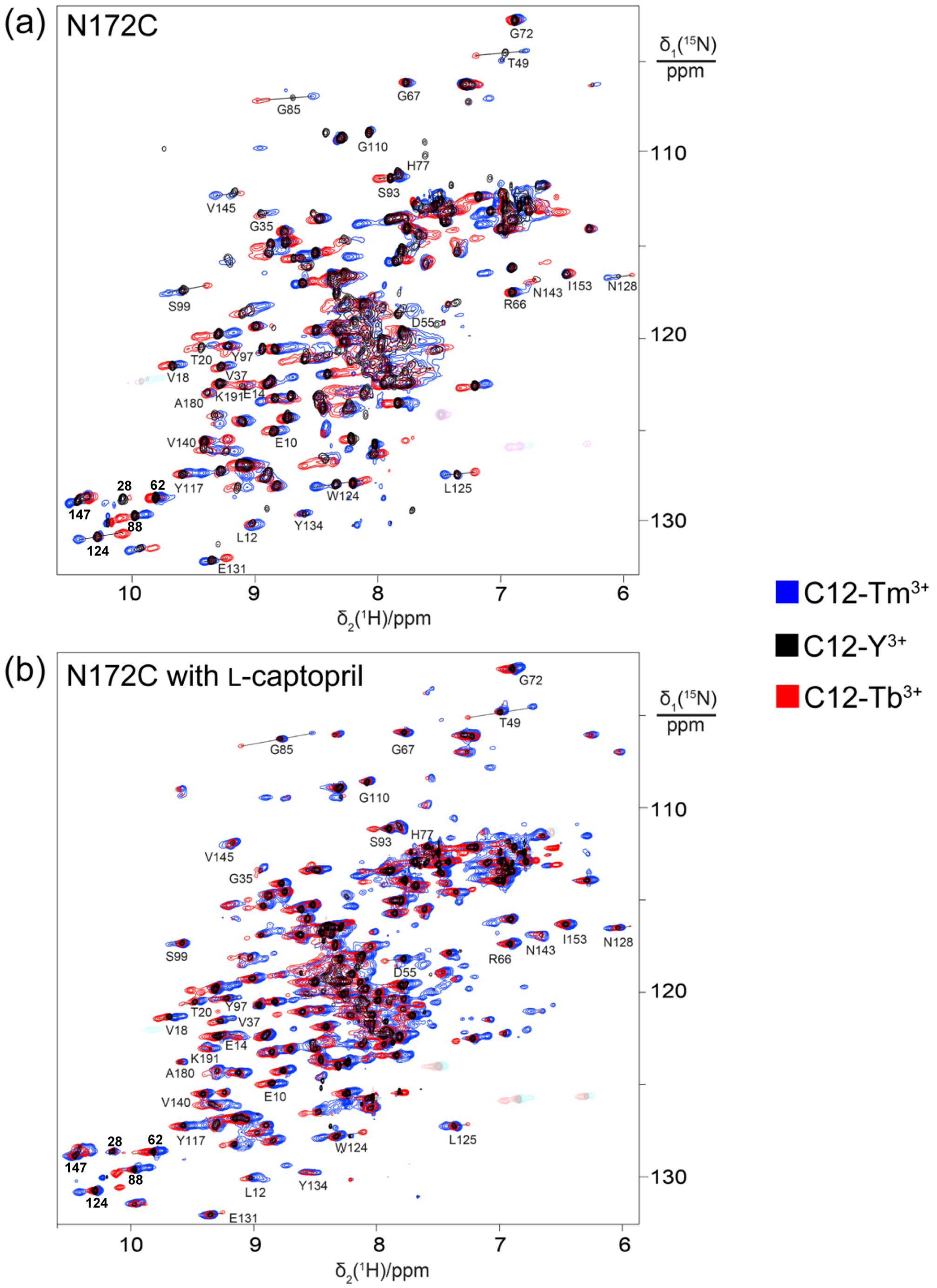

Figure S7. Superimpositions of [^{15}N,^{1}H]-HSQC spectra recorded of 0.2 mM solutions of uniformly ^{15}N-labelled IMP-1 N172C ligated with the C12 tag containing Tb^{+3} (red), Tm^{3+} (blue) or Y^{3+} (black) ions. Cross-peaks of tryptophan indole NH groups are labelled in bold with the residue number only. (a) Spectra of free protein. (b) Spectra in the presence of 1.5-fold excess of captopril.

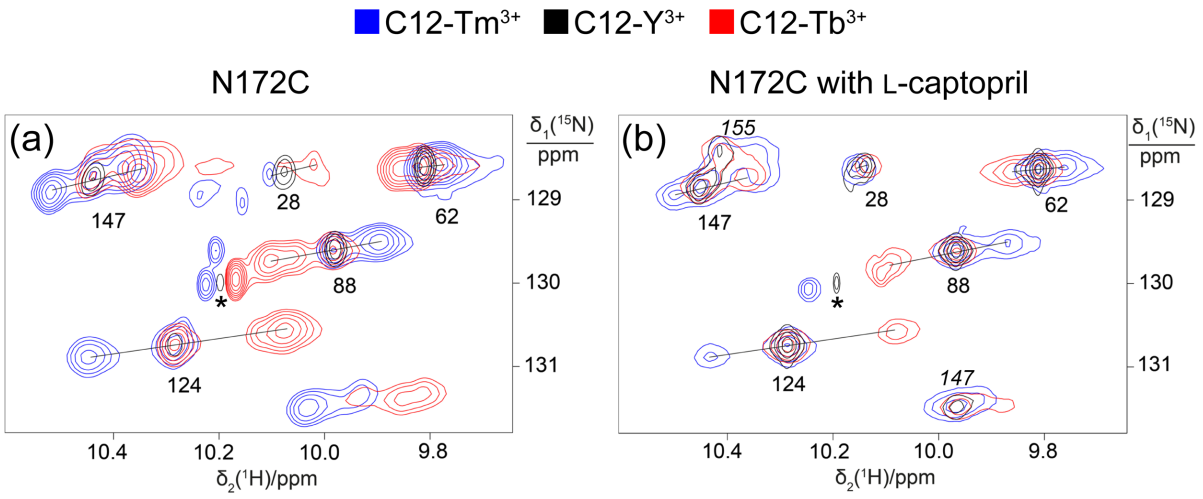

Figure S8. Spectral region of the [^{15}N,^{1}H]-HSQC spectra of Figure S6 showing the indole $N^{\varepsilon1}$–$H^{\varepsilon1}$ cross-peaks of the tryptophan residues in the (a) absence and (b) presence of captopril. The PCSs of Trp28 $H^{\varepsilon1}$ are much smaller than for the same mutant (N172C) labelled with the C2 tag (Figure S5b). Stars mark cross-peaks in the diamagnetic sample (made with C12-Y^{3+} tag) attributed to sample degradation. Cross-peaks in the diamagnetic sample assigned to tryptophan indole NH groups and backbone amides are labelled in bold and italics, respectively.

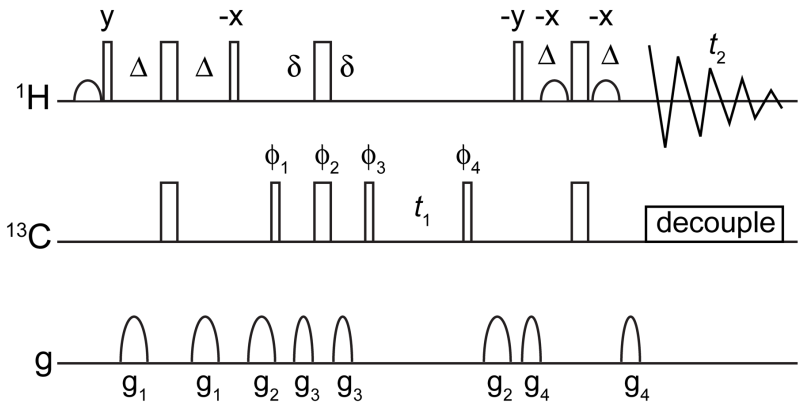

Figure S9. Pulse sequence of [^{13}C,^{1}H]-HSQC with S^{3}E filter designed to select the low-field TROSY component in the ^{13}C dimension. Narrow and wide rectangles denote 90° and 180° pulses, respectively. Round pulse shapes indicate water-selective 90° pulses of 0.9 ms duration with the shape of the centre lobe of a sinc function. Pulse phases are x unless indicated otherwise. Delays: $\Delta = 2\delta = 1/(4^1J_{HC}) = 1.56$ ms. Pulsed field gradients: g_1 = 1 ms at 4 G/cm; g_2 = 1 ms at 19 G/cm; g_3 = 0.4 ms at 3.5 G/cm; g_4 = 0.4 ms at 12.5 G/cm. Phase cycle: ϕ_1 = x,-x; ϕ_2 = 22.5° relative to x; ϕ_3 = y,y,-y,-y; ϕ_4 = x; receiver = x,-x. Quadrature detection in the F_1 dimension is achieved by decrementing the phase ϕ_4 in the State-TPPI manner.

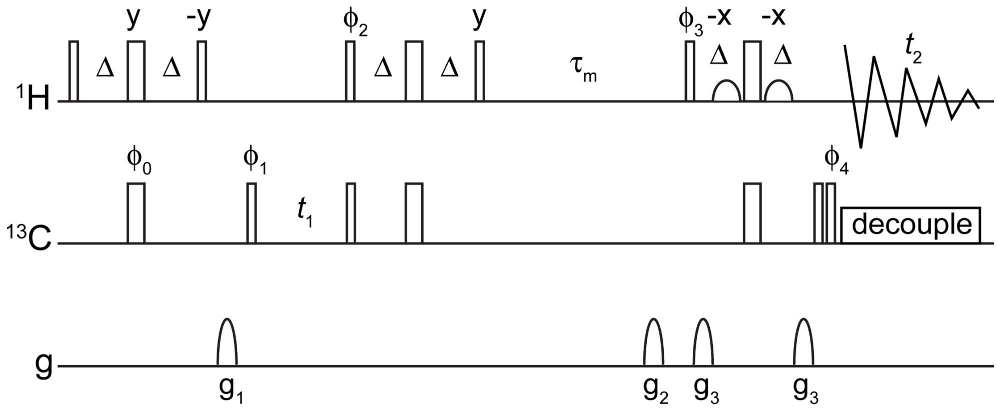

Figure S10. Pulse sequence of [^{13}C,^{1}H]-HSQC with NOE relay and without decoupling in the ^{13}C-dimension. The pulse sequence detects both doublet components in the ^{13}C-dimension, but only the narrow TROSY component is readily detected in large proteins. Narrow and wide rectangles denote 90° and 180° pulses, respectively. Round pulse shapes indicate water-selective 90° pulses of 1 ms duration with the shape of the centre lobe of a sinc function. Pulse phases are x unless indicated otherwise. Delays: $\Delta = 1/(4^1J_{HC}) = 1.56$ ms. Pulsed field gradients: g_1 = 0.6 ms at 25 G/cm; g_2 = 0.6 ms at 10 G/cm; g_3 = 0.6 ms at 40 G/cm. Phase cycle: ϕ_1 = x,-x; ϕ_2 = 2(-x),2(x); ϕ_3 = 4(x),4(-x); ϕ_4 = 8(x),8(-x); receiver = x,-x,-x,x,-x,x,x,-x. ϕ_0 and ϕ_1 are incremented in the State-TPPI manner.

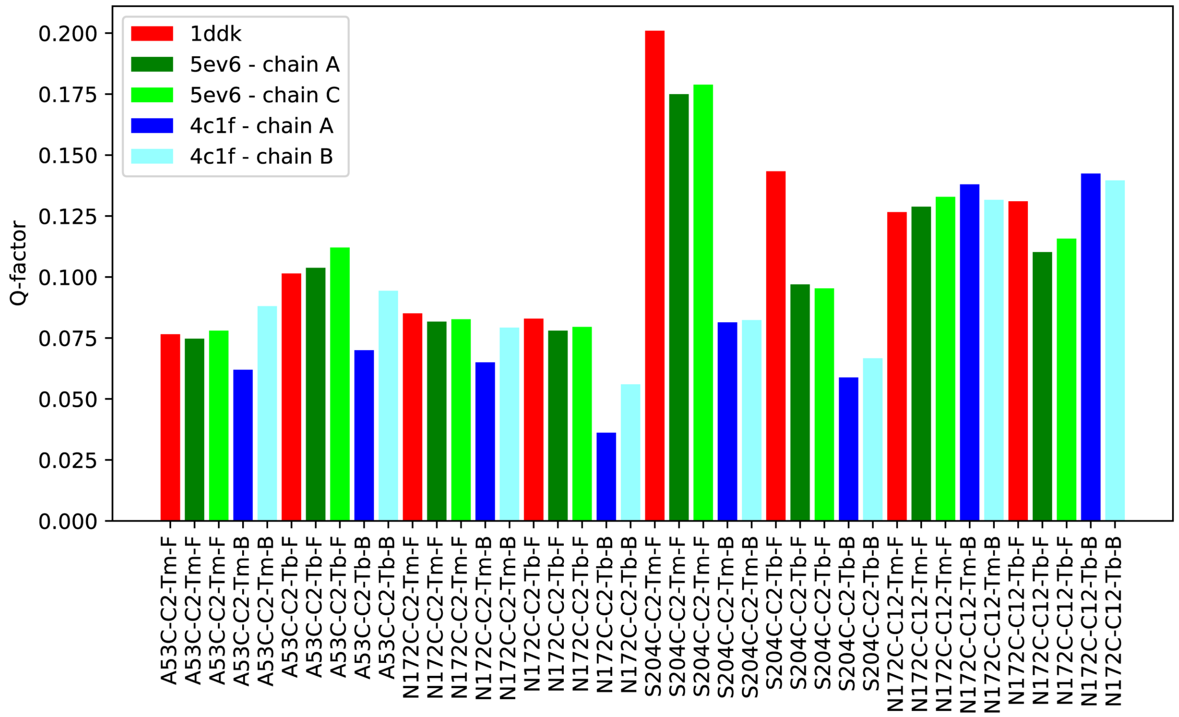

Figure S11. *Q* factors of $\Delta\chi$-tensor fits to ^{1}H PCSs measured of backbone amides for different PDB structures using the program Paramagpy (Orton et al., 2020). Hydrogen atoms were added to the crystal structures using the program PyMOL (Schrödinger, LLC, 2015). The x-axis identifies the dataset used; for example, A53C-C2-Tm-F used the ^{1}H PCSs of backbone amides in the A53C mutant ligated with the C2-Tm^{3+} tag. "F" and "B" refer to the free protein (in the absence of captopril) and bound protein (i.e. in the presence of captopril).

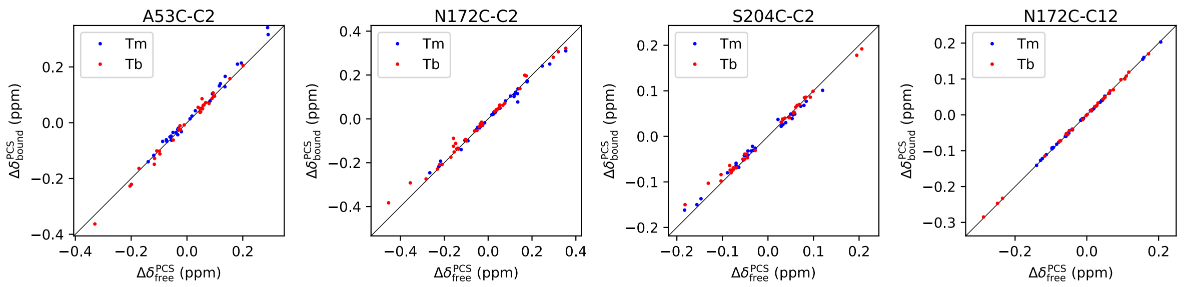

Figure S12. Correlations of ^{1}H PCSs measured of backbone amides in the presence versus the absence of captopril.

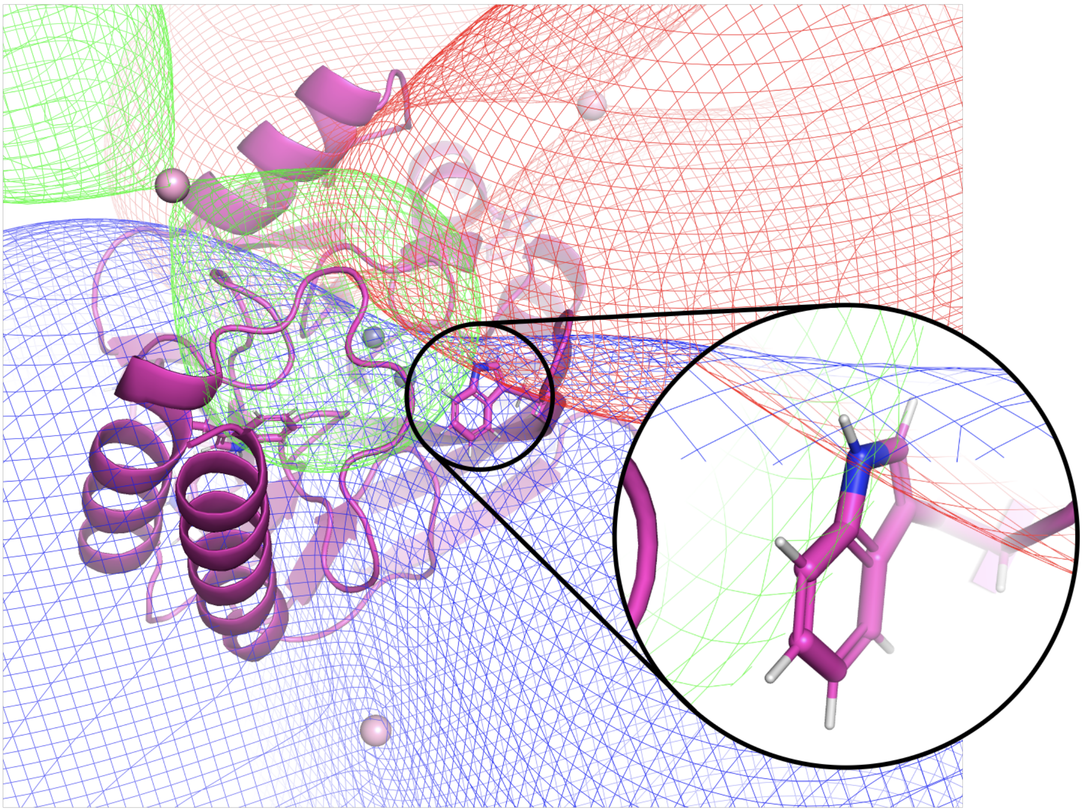

Figure S13. PCS isosurfaces plotted for the IMP-1 mutants A53C, N172C and S204C ligated with C2-Tb^{3+} tags shown in red, green and blue, respectively, at a contour level equal to the experimental PCS measured for the sidechain $H^{\varepsilon 1}$ atom of Trp28. Data recorded in the absence of captopril and plotted on the structure 5EV6. The protein is shown in a cartoon representation (magenta) with the Zn^{2+} ions in the active site shown as grey spheres. The side-chain of Trp28 is highlighted by a stick representation. The figure shows that the three PCS isosurfaces intersect next to the Trp28 $H^{\varepsilon 1}$ atom.

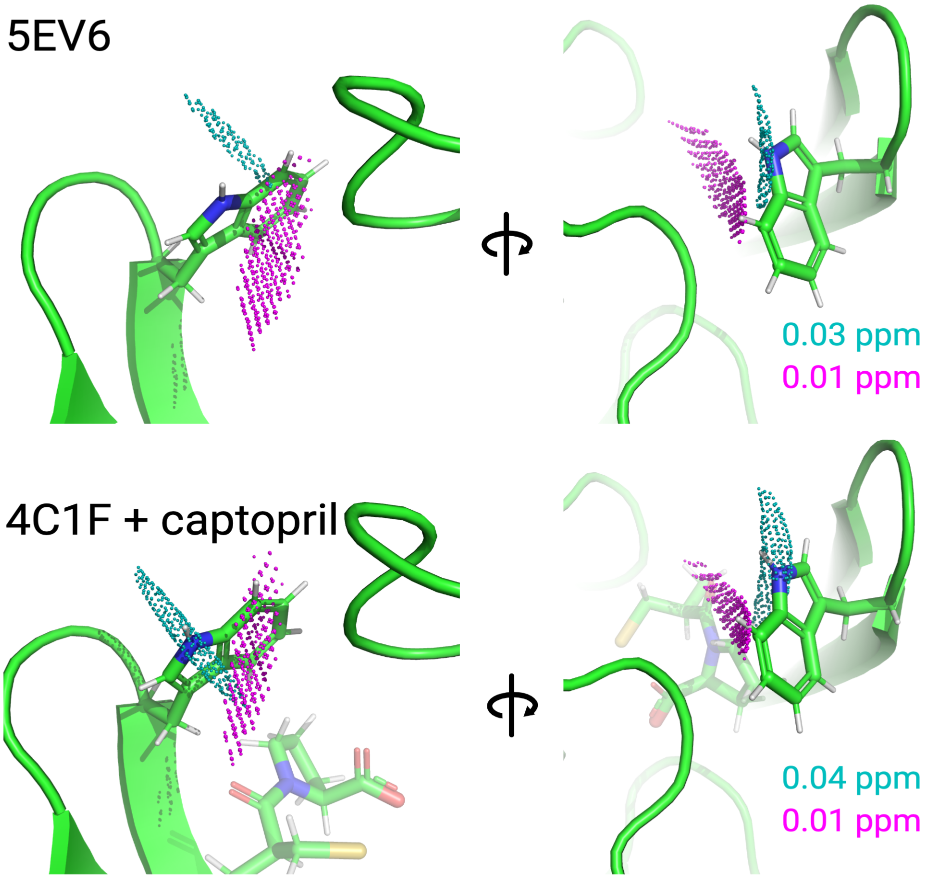

Figure S14. Localisation spaces of the $H^{\zeta2}$ and $H^{\varepsilon1}$ atoms of Trp28 defined by the PCSs obtained with Tm^{3+} tags. Data were obtained for the IMP-1 mutants A53C, N172C and S204C ligated with C2-Tm^{3+} tags, both in the absence and presence of captopril. The localisation space of the $H^{\varepsilon1}$ atom was restricted further by a fourth PCS obtained for the mutant N172C with the C12-Tm^{3+} tag. Pink and cyan points trace the localisation spaces of the $H^{\zeta2}$ and $H^{\varepsilon1}$ atoms, respectively. The maximal PCS RMSD values defining the boundaries of the localisation spaces are indicated in ppm. The top panel depicts the localisation spaces determined of the free protein, plotted on the crystal structure 5EV6 and depicted in two different orientations. The bottom panel depicts the localisation spaces determined in the presence of captopril, plotted on the crystal structure 4C1F. The localisation spaces are shown in two different orientations differing by a 180° rotation (about a tilted axis).

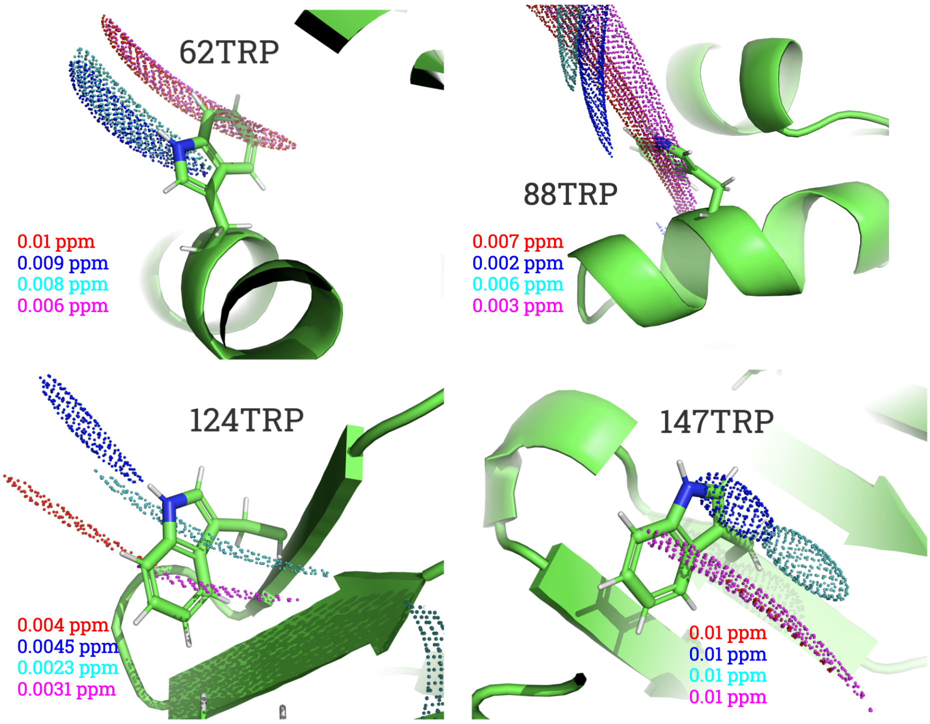

Figure S15. Localisation spaces of the $H^{\zeta 2}$ and $H^{\varepsilon 1}$ atoms of Trp62, Trp88, Trp124 and Trp147 in the absence of an inhibitor, plotted on the structure 5EV6. Red and blue points trace the localisation spaces of the $H^{\zeta 2}$ (red) and $H^{\varepsilon 1}$ (blue) atoms derived from PCS obtained with Tb^{3+} tags. PCS RMSD cutoffs used to trace the boundaries of the localisation spaces are indicated with the same colours. Corresponding localization spaces obtained with Tm^{3+} tags are shown in pink and cyan. The localization spaces are less well-defined than for Trp28, as some of the tagging sites produced very small PCSs for these atoms.

Table S1. Pseudocontact shifts measured in ppm for backbone amide protons of IMP-1 cysteine mutants ligated with lanthanoid tags.[a]

	A53C-C2		N172C-C2		S204C-C2		N172C-C12	
Residue	Tb	Tm	Tb	Tm	Tb	Tm	Tb	Tm
4LEU	-0.344	0.289	-0.135	0.102	-	-0.044	-	-
8LYS	-0.428	0.365	-0.148	0.120	0.234	-0.188	0.110	-0.097
10GLU	-	-	-0.106	0.083	-	-	-	-
12LEU	-	-	-0.069	0.061	-	-	0.013	-0.017
14GLU	-0.029	0.036	-0.042	0.033	0.321	-0.244	-	-0.009
15GLY	-0.049	0.054	-0.030	0.025	-	-0.183	0.011	-0.013
17TYR	-0.097	0.091	-0.066	0.058	-	-	-	-
18VAL	-0.117	0.120	-0.103	0.081	-	-	0.022	-0.060
19HIS	-0.199	0.181	-0.155	0.125	0.245	-0.207	-	-
20THR	-0.330	0.291	-0.162	0.127	-	-	0.106	-0.097
22PHE	-	-	-0.231	0.178	-	-0.089	-	-
24GLU	-	-	-0.294	0.219	-	-0.027	-	-
25VAL	-	-	-0.417	0.310	-	-0.009	-	-
31VAL	-0.111	0.125	-0.389	0.291	-	-0.043	-	-
34HIS	-	-	-	-	-	-	-	-0.093
35GLY	-0.204	0.195	-0.222	0.178	0.195	-0.147	0.095	-0.113
36LEU	-	-	-	-	0.119	-0.082	0.031	-0.078
37VAL	-0.116	0.116	-0.158	0.135	0.206	-0.156	0.028	-0.058
40VAL	-0.008	0.015	-0.094	0.078	0.069	-0.054	-	-
42ALA	-	-	-	-	0.061	-0.045	-	-
44ALA	-	-	-	-	0.066	-0.040	-	-
45TYR	-0.017	0.034	-0.025	0.023	-	-	-	-
46LEU	-	-	-0.031	0.034	0.056	-0.046	-	-
47ILE	-0.083	0.093	-0.098	0.088	0.080	-0.064	-	-
48ASP	-0.021	0.056	-0.143	0.125	0.031	-0.024	0.074	-0.124
49THR	-0.099	0.137	-0.210	0.177	0.029	-0.034	-	-
52THR	-	-	-0.170	0.136	0.058	-0.048	-	-
55ASP	-	-	-0.099	0.079	0.064	-0.052	-	-
56THR	-	-	-	-	0.069	-0.057	-	-
59LEU	-	-	-	-	0.106	-0.083	-	-
61THR	-	-	-0.041	0.033	0.090	-0.069	-	-
64VAL	-0.229	0.179	-0.026	0.022	0.093	-0.070	0.061	-0.041
66ARG	-0.172	0.136	-0.026	0.017	0.099	-0.077	-	-

67GLY	−0.108	0.083	–	–	0.083	−0.070	0.026	−0.040
70ILE	0.040	−0.020	–	–	0.056	−0.044	0.047	−0.050
71LYS	0.061	−0.033	–	–	0.047	−0.037	–	–
72GLY	0.095	−0.055	–	–	0.037	−0.033	0.001	−0.040
73SER	–	–	–	–	0.018	−0.019	–	–
74ILE	–	–	−0.051	0.054	0.015	−0.017	–	–
76SER	–	–	−0.283	0.247	–	–	0.045	−0.140
77HIS	0.050	–	−0.465	0.409	−0.033	0.014	–	–
78PHE	0.083	−0.054	–	–	−0.028	0.022	–	–
82SER	–	–	−0.537	0.435	–	–	–	–
83THR	–	–	−0.373	0.302	–	–	–	–
84GLY	–	–	−0.256	0.212	0.024	−0.023	–	–
85GLY	–	–	−0.138	0.119	0.032	−0.028	–	–
88TRP	–	–	–	–	0.029	−0.025	–	–
89LEU	–	–	–	–	0.034	−0.026	–	–
90ASN	–	–	0.042	−0.030	–	–	0.172	−0.129
92ARG	–	–	0.037	−0.028	0.022	−0.017	0.117	−0.084
93SER	–	–	0.051	−0.039	0.014	−0.013	0.051	−0.057
94ILE	–	–	0.038	−0.025	0.022	−0.021	0.065	−0.051
97TYR	0.201	−0.139	–	–	–	–	–	–
99SER	0.090	−0.058	–	–	−0.030	0.018	−0.249	0.159
104GLU	0.067	−0.061	–	–	−0.019	0.010	–	–
106LEU	0.094	−0.087	–	–	−0.012	0.006	–	–
109ASP	0.054	−0.074	–	–	–	–	–	–
110GLY	−0.047	–	–	–	–	–	−0.002	−0.016
114ALA	–	–	0.174	−0.123	–	–	–	–
117SER	0.154	−0.119	0.234	−0.182	–	–	–	–
118PHE	0.099	−0.072	0.297	−0.225	–	–	−0.235	0.206
119SER	0.058	−0.050	0.354	−0.266	−0.031	0.021	–	–
120GLY	–	–	–	–	−0.051	0.031	−0.011	0.043
123TYR	0.036	−0.019	0.190	−0.131	−0.081	0.055	–	–
124TRP	0.047	−0.033	0.145	−0.106	−0.055	0.039	–	–
125LEU	0.047	−0.028	0.076	−0.052	−0.047	0.029	−0.076	0.050
128ASN	–	–	0.067	−0.048	−0.069	0.053	−0.058	0.018
129LYS	–	–	0.044	−0.029	−0.051	0.033	−0.053	0.020
130ILE	–	–	0.040	−0.026	−0.035	0.028	−0.040	0.037
131GLU	–	–	0.030	−0.016	−0.083	0.059	−0.075	0.041

132VAL	0.030	-0.018	0.102	-0.069	-	-	-	-
133PHE	-	-	0.070	-0.028	-0.116	0.077	-	-
134TYR	0.022	0.014	0.320	-0.217	-0.103	0.072	-	-
140THR	0.034	-0.018	-	-	-0.077	0.050	-	-
142ASP	0.049	-0.031	-	-	-0.045	0.030	-	-
143ASN	0.041	-0.023	-	-	-0.076	0.046	-	-
144VAL	0.029	-0.008	-0.351	0.336	-0.084	0.055	-	-
145VAL	-	-	-0.025	0.059	-0.131	0.084	-0.288	0.156
147TRP	-	-	-	-	-0.103	0.079	-0.117	0.063
148LEU	-0.015	0.023	-0.018	0.025	-	-	-0.049	0.010
150GLU	-	-	-	-	-0.089	0.069	-	-
151ARG	-0.009	0.014	-	-	-0.066	0.053	-	-
153ILE	-	-	-0.032	0.030	-0.182	0.120	-0.057	0.013
154LEU	-0.029	0.036	-0.080	0.073	-	-	-	-
156GLY	-0.048	0.047	-	-	-	-	-	-
157GLY	-0.050	0.051	-	-	-	-	-	-
159PHE	-	-	-	-	-0.155	0.121	-	-
160ILE	-0.023	0.030	-	-	-	0.190	-	-
161LYS	-0.025	0.018	-	-	-	-	-	-
163TYR	-0.041	0.028	-	-	-	-	-	-
169GLY	0.079	-0.057	-	-	-0.010	0.017	-	-
171ALA	0.056	-0.039	-	-	-0.025	0.015	-	-
178LYS	-	-	-	-	-0.045	0.031	-	-
180ALA	-	-	0.167	-0.092	-0.119	0.085	-	-
181LYS	-	-	-	-	-0.127	0.074	-	-
188GLY	-	-	0.053	-0.035	-	-	-	-
189LYS	-	-	0.031	-0.016	-	-	-0.073	-
191LYS	-0.020	0.020	-0.036	0.027	-	-	-	-
192LEU	-	-	-0.062	0.053	-	-	-	-
193VAL	-0.060	0.052	-0.122	-	-	-	-	-
194VAL	-0.066	0.056	-0.174	0.145	-	-	-	-
198SER	-0.108	0.091	-	-	-	-	-	-
201GLY	-	-	-0.176	0.141	-	-	-	-
204SER	-	-	-0.089	0.073	-	-	-	-
210LEU	-0.010	0.011	-0.158	0.134	-	-	-	-
211GLU	-	-	-0.148	0.118	-	-	-	-
213ALA	-	-	-0.355	0.281	-	0.088	-	-

216GLY	–	–	−0.454	0.354	0.058	−0.022	–	–
217LEU	–	–	–	–	0.044	−0.027	–	–

[a] The cross-peaks of amide protons showed full linewidths at half-height of up to about 70 Hz in paramagnetic samples, while signal-to-noise ratios (S/N) typically were at least 6:1. Estimating the uncertainty of peak position as a quarter of 70 Hz yields a PCS uncertainty of 0.02 ppm. The $\Delta\chi$-tensor fits (Figure 4) suggest that actual uncertainties were of this order of magnitude or smaller. An accurate estimate of uncertainties is complicated by the sensitivity of the chemical shifts (in particular of amide protons) to minor differences in sample conditions between paramagnetic and diamagnetic samples, the impact of which is difficult to predict.

Table S2. Pseudocontact shifts measured in ppm for backbone amide protons of IMP-1 cysteine mutants ligated with lanthanoid tags and in the presence of 1.5-fold excess of the inhibitor captopril.

	A53C-C2		N172C-C2		S204C-C2		N172C-C12	
Residue	Tb	Tm	Tb	Tm	Tb	Tm	Tb	Tm
4LEU	−0.414	0.341	−0.138	0.104	0.054	−0.032	–	–
8LYS	–	–	–	–	–	–	0.108	−0.092
12LEU	–	–	–	–	–	–	0.014	−0.015
14GLU	–	–	–	–	–	–	–	−0.010
15GLY	−0.062	0.052	−0.015	0.026	0.236	−0.162	0.011	−0.011
16VAL	–	–	–	0.081	–	–	–	–
17TYR	−0.112	0.104	–	–	–	–	–	–
18VAL	−0.149	0.140	−0.094	–	–	–	0.023	−0.060
19HIS	−0.221	0.210	−0.151	0.121	–	–	–	–
20THR	−0.363	0.316	–	–	–	–	0.100	−0.095
22PHE	–	–	−0.229	0.170	0.098	−0.080	–	–
34HIS	–	–	–	–	–	–	–	−0.091
35GLY	−0.227	0.214	−0.209	0.173	0.178	−0.137	0.098	−0.111
36LEU	–	–	–	–	–	–	0.033	−0.076
37VAL	−0.128	0.132	−0.089	0.077	0.192	−0.150	0.026	−0.060
40VAL	–	–	–	–	0.070	−0.052	–	–
42ALA	–	–	–	–	0.064	−0.043	–	–
45TYR	–	–	−0.023	0.021	0.075	–	–	–
46LEU	–	–	−0.031	0.031	0.051	−0.044	–	–
47ILE	–	–	–	–	0.085	−0.068	–	–
48ASP	–	–	−0.137	0.118	–	–	0.070	−0.122
49THR	−0.102	0.166	−0.208	0.169	0.031	−0.022	–	–
52THR	–	–	−0.175	0.137	0.052	−0.040	–	–

55ASP	–	–	-0.101	0.082	0.068	-0.042	–	–
61THR	–	–	-0.036	0.031	–	–	–	–
64VAL	–	–	-0.026	0.022	0.086	-0.064	0.060	-0.040
65GLU	-0.228	0.167	–	–	–	–	–	–
66ARG	-0.164	0.129	-0.025	0.019	0.099	-0.073	–	–
67GLY	-0.101	0.078	-0.015	0.011	0.086	-0.059	0.024	-0.040
70ILE	0.055	-0.032	–	–	0.053	-0.041	0.045	-0.051
71LYS	0.064	-0.043	–	–	0.041	-0.032	–	–
72GLY	0.095	-0.063	–	–	0.040	-0.031	0.001	-0.042
74ILE	–	–	-0.053	0.049	–	–	–	–
76SER	–	–	-0.274	0.241	–	–	0.044	-0.141
78PHE	–	–	–	–	-0.031	0.037	–	–
82SER	-0.068	0.092	–	–	–	–	–	–
85GLY	–	–	-0.134	0.111	0.038	-0.026	–	–
90ASN	–	–	–	–	–	–	0.170	-0.127
92ARG	–	–	0.042	-0.024	–	–	0.119	-0.082
93SER	–	–	0.053	-0.041	–	–	0.049	-0.055
94ILE	–	–	0.039	-0.029	–	–	0.067	-0.050
97TYR	0.205	-0.140	–	–	–	–	–	–
99SER	0.087	-0.052	–	–	–	–	-0.247	0.160
104GLU	0.072	-0.051	–	–	–	–	–	–
106LEU	0.107	-0.067	–	–	–	–	–	–
109ASP	0.086	-0.061	–	–	–	–	–	–
110GLY	–	–	–	–	–	–	-0.002	-0.015
114ALA	–	–	0.196	-0.140	–	–	–	–
117SER	0.158	-0.117	–	–	–	–	–	–
118PHE	0.095	-0.067	0.281	-0.218	–	–	-0.233	0.203
119SER	0.062	-0.035	0.322	-0.246	–	–	–	–
120GLY	–	–	–	–	-0.050	0.025	-0.013	0.040
123TYR	–	–	–	–	-0.080	0.047	–	–
124TRP	0.051	-0.024	0.138	-0.098	-0.050	0.030	–	–
125LEU	0.037	-0.025	0.071	-0.047	-0.039	0.022	-0.074	0.052
128ASN	–	–	0.062	-0.039	-0.069	0.039	-0.055	0.016
129LYS	–	–	0.045	-0.024	-0.039	0.026	-0.055	0.018
130ILE	–	–	–	–	–	–	-0.040	0.035
131GLU	–	–	–	–	-0.074	0.049	-0.073	0.040
134TYR	–	–	0.306	-0.193	-0.084	0.066	–	–

140THR	–	–	–	–	−0.074	0.049	–	–
142ASP	0.039	−0.020	–	–	−0.047	0.031	–	–
143ASN	–	–	–	–	−0.069	0.042	–	–
144VAL	–	–	–	–	−0.064	0.048	–	–
145VAL	–	–	–	–	−0.103	0.077	−0.285	0.155
147TRP	–	–	–	–	−0.098	0.068	−0.115	0.060
148LEU	–	–	–	–	–	–	−0.044	0.012
153ILE	–	0.037	−0.023	0.028	−0.150	0.101	−0.052	0.011
154LEU	−0.023	–	–	–	–	–	–	–
160ILE	−0.026	0.043	–	–	–	–	–	–
161LYS	−0.011	0.024	–	–	–	–	–	–
169GLY	0.069	−0.048	–	–	–	–	–	–
171ALA	0.050	−0.035	–	–	–	–	–	–
180ALA	–	–	0.199	−0.100	–	–	–	–
188GLY	–	–	0.063	−0.035	–	–	–	–
189LYS	–	–	0.034	−0.018	–	–	−0.072	–
191LYS	–	–	−0.023	0.032	–	–	–	–
192LEU	–	–	−0.055	0.049	–	–	–	–
210LEU	−0.008	0.014	−0.125	0.115	–	–	–	–
211GLU	–	–	−0.112	0.102	–	–	–	–
213ALA	–	–	−0.292	0.250	–	–	–	–
216GLY	–	–	−0.383	0.310	–	–	–	–
217LEU	0.018	−0.005	–	–	–	–	–	–

Table S3. Pseudocontact shifts measured in ppm for the tryptophan $H^{\zeta2}$ protons in the IMP-1 cysteine mutants ligated with C2-Tb^{3+} or C2-Tm^{3+} tags.

	A53C-C2		N172C-C2		S204C-C2		N172C-C12	
Residue	Tb	Tm	Tb	Tm	Tb	Tm	Tb	Tm
28TRP	0.048	−0.027	−1.228	0.918	−0.046	0.038	–	–
62TRP	−0.228	0.182	−0.056	0.046	0.227	−0.169	–	–
88TRP	0.392	−0.460	−0.014	0.014	0.038	−0.030	–	–
124TRP	0.018	−0.009	0.088	−0.058	−0.085	0.060	–	–
147TRP	−0.004	0.009	0.028	−0.010	−0.318	0.229	–	–
176TRP	0.003	0.004	–	–	−0.124	0.110	–	–

Table S4. Pseudocontact shifts measured in ppm for the tryptophan $H^{\varepsilon1}$ protons in the IMP-1 cysteine mutants ligated with C2 and C12 lanthanoid tags.

	A53C-C2		N172C-C2		S204C-C2		N172C-C12	
Residue	Tb	Tm	Tb	Tm	Tb	Tm	Tb	Tm
28TRP	0.078	-0.049	-1.102	0.851	-0.040	0.028	-0.055	0.150
62TRP	-0.305	0.248	-0.057	0.047	0.175	-0.136	0.044	-0.042
88TRP	-	-	-0.010	0.010	0.029	-0.023	0.125	-0.087
124TRP	0.026	-0.018	0.104	-0.069	-0.068	0.049	-	-
147TRP	-0.001	0.003	0.049	-0.024	-0.209	0.150	-0.086	0.073
176TRP	0.004	0.002	-	-	-0.140	0.128	-	-

Table S5. Pseudocontact shifts measured in ppm for the tryptophan $H^{\zeta2}$ protons in the IMP-1 cysteine mutants ligated with C2 lanthanoid tags and in the presence of captopril.

	A53C-C2		N172C-C2		S204C-C2		N172C-C12	
Residue	Tb	Tm	Tb	Tm	Tb	Tm	Tb	Tm
28TRP	0.073	-0.057	-1.327	0.952	-0.038	0.033	-	-
62TRP	-0.239	0.199	-0.057	0.045	0.219	-0.158	-	-
88TRP	0.138	-0.169	-0.010	0.009	0.039	-0.028	-	-
124TRP	0.012	-0.004	0.090	-0.059	-0.100	0.069	-	-
147TRP	-0.008	0.011	0.029	-0.014	-0.330	0.219	-	-
176TRP	0.002	0.003	-	-	-0.132	0.104	-	-

Table S6. Pseudocontact shifts measured in ppm for the tryptophan $H^{\varepsilon1}$ protons in the IMP-1 cysteine mutants ligated with lanthanoid tags and in the presence of captopril.

	A53C-C2		N172C-C2		S204C-C2		N172C-C12	
Residue	Tb	Tm	Tb	Tm	Tb	Tm	Tb	Tm
28TRP	0.108	-0.089	-1.136	0.798	-0.025	0.022	0.001	0.020
62TRP	-0.319	0.268	-0.059	0.046	0.176	-0.129	0.057	-0.066
88TRP	-	-	-0.005	0.002	0.033	-0.025	0.147	-0.117
124TRP	0.020	-0.010	0.118	-0.079	-0.080	0.050	-0.199	0.147
147TRP	-0.002	0.007	0.049	-0.025	-0.210	0.140	-0.085	0.029
176TRP	0.003	0.002	-	-	-0.154	0.118	-	-

Table S7. Δχ tensors fitted to the chain A of the structures 5EV6 and 4C1F using the ^{1}H PCSs of backbone amides.

PDB ID	captopril	Site	Tag	Ion	$\Delta\chi_{ax}$[a]	$\Delta\chi_{rh}$[a]	x (Å)	y (Å)	z (Å)	α (°)	β (°)	γ (°)	*d* (Å)[b]	*Q*
5EV6	no	A53C	C2	Tm	9.8	4.6	57.396	106.347	30.973	150	130	75	8.8	0.07
5EV6	no	A53C	C2	Tb	-12.6	-5.9	57.396	106.347	30.973	156	130	79	8.8	0.10
5EV6	no	N172C	C2	Tm	13.8	3.4	40.922	77.867	25.436	20	32	173	8.2	0.08
5EV6	no	N172C	C2	Tb	-17.9	-4.4	40.922	77.867	25.436	21	30	173	8.2	0.08
5EV6	no	S204C	C2	Tm	-6.3	-2.7	67.764	68.485	49.864	52	104	71	8.3	0.18
5EV6	no	S204C	C2	Tb	9.0	4.1	67.764	68.485	49.864	47	103	65	8.3	0.10
5EV6	no	N172C	C12	Tm	-10.5	-5.1	43.971	85.261	25.852	57	54	106	12.1	0.13
5EV6	no	N172C	C12	Tb	13.5	6.4	43.971	85.261	25.852	61	52	112	12.1	0.11
4C1F	yes	A53C	C2	Tm	10.4	5.5	2.684	31.225	13.791	167	19	114	8.9	0.06
4C1F	yes	A53C	C2	Tb	-12.8	-6.2	2.684	31.225	13.791	176	17	109	8.9	0.07
4C1F	yes	N172C	C2	Tm	13.8	4.0	10.430	1.144	23.907	43	136	40	8.8	0.07
4C1F	yes	N172C	C2	Tb	-17.9	-5.3	10.430	1.144	23.907	43	134	41	8.8	0.04
4C1F	yes	S204C	C2	Tm	-7.1	-0.9	21.056	0.080	15.721	119	93	2	9.4	0.08
4C1F	yes	S204C	C2	Tb	9.6	1.8	21.056	0.080	15.721	121	94	12	9.4	0.06
4C1F	yes	N172C	C12	Tm	-9.3	-4.3	9.353	8.135	21.272	85	118	30	11.1	0.14
4C1F	yes	N172C	C12	Tb	11.7	5.2	9.353	8.135	21.272	82	115	38	11.1	0.14

[a] In units of 10^{-32} m^3. Coordinates and Euler angles are reported with respect to the respective PDB coordinates. Euler angles are given in "ZYZ" convention. See also Fig. S11 for the *Q* factors of the fits.

[b] Distance *d* between Δχ tensor origin and C$^{\beta}$ atom of the mutated residue.

Table S8. Uncertainty ranges associated with the Δχ-tensor parameters of Table S7.[a]

PDB ID	captopril	Site	Tag	Ion	$\Delta\chi_{ax}$	$\Delta\chi_{rh}$	x (Å)	y (Å)	z (Å)	α (°)	β (°)	γ (°)
5EV6	no	A53C	C2	Tm	0.4	0.2	0.15	0.19	0.17	1	1	1
5EV6	no	A53C	C2	Tb	0.5	0.3	0.15	0.19	0.17	1	0	0
5EV6	no	N172C	C2	Tm	0.2	0.3	0.13	0.27	0.20	2	1	24
5EV6	no	N172C	C2	Tb	0.4	0.4	0.13	0.27	0.20	2	1	3
5EV6	no	S204C	C2	Tm	1.8	1.0	0.31	0.82	0.47	5	11	5
5EV6	no	S204C	C2	Tb	1.0	0.4	0.31	0.82	0.47	1	2	3
5EV6	no	N172C	C12	Tm	1.2	1.0	0.48	0.36	0.36	3	1	4
5EV6	no	N172C	C12	Tb	1.1	0.7	0.48	0.36	0.36	2	1	4
4C1F	yes	A53C	C2	Tm	0.4	0.3	0.39	0.23	0.21	3	1	4
4C1F	yes	A53C	C2	Tb	0.5	0.4	0.39	0.23	0.21	60	51	10
4C1F	yes	N172C	C2	Tm	0.4	0.3	0.16	0.42	0.15	2	1	2
4C1F	yes	N172C	C2	Tb	0.3	0.3	0.16	0.42	0.15	2	1	1
4C1F	yes	S204C	C2	Tm	0.2	0.2	0.34	0.24	0.21	1	1	0
4C1F	yes	S204C	C2	Tb	0.3	0.3	0.34	0.24	0.21	1	1	0
4C1F	yes	N172C	C12	Tm	1.2	1.1	0.78	0.34	0.30	3	3	2
4C1F	yes	N172C	C12	Tb	1.7	1.0	0.78	0.34	0.30	2	4	9

[a] Uncertainty ranges determined by randomly omitting 20% of the PCSs over 50 samples. The values in the table correspond to half of the full uncertainty ranges. x, y, z coordinates in Table S7 contain

redundant significant digits to match the PDB format. Units of $\Delta\chi_{ax}$ and $\Delta\chi_{rh}$ as in Table S7.

References

Carruthers, T. J.: Paramagnetism & Structural Biology: Biochemical & Biophysical Analysis of IMP-1 Metallo-β-lactamase, PhD thesis, The Australian National University, Canberra, 221 pp., 2014.

Orton, H. W., Huber, T., and Otting, G.: Software for fitting magnetic susceptibility tensors using paramagnetic effects measured in NMR spectra, Magn. Reson., 1, 1–12, https://doi.org/10.5194/mr-1-1-2020, 2020.

Schrödinger, LLC: The PyMOL molecular graphics system, version 1.8, 2015.
